# SHOC1 is a ERCC4-(HhH)_2_-like protein, integral to the formation of crossover recombination intermediates during mammalian meiosis

**DOI:** 10.1371/journal.pgen.1007381

**Published:** 2018-05-09

**Authors:** Michel F. Guiraldelli, Anna Felberg, Luciana P. Almeida, Aniruddha Parikh, Rodrigo O. de Castro, Roberto J. Pezza

**Affiliations:** 1 Oklahoma Medical Research Foundation, Oklahoma City, Oklahoma, United States of America; 2 Department of Cell Biology, University of Oklahoma Health Science Center, Oklahoma City, Oklahoma, United States of America; National Cancer Institute, UNITED STATES

## Abstract

Chromosome segregation errors during meiosis result in the formation of aneuploid gametes and are the leading cause of pregnancy loss and birth defects in humans. Proper chromosome segregation requires pairwise associations of maternal and paternal homologous chromosomes. Chiasmata, which are the cytological manifestations of crossovers (COs), provide a physical link that holds the homologs together as a pair, facilitating their orientation on the spindle at meiosis I. Although CO-promoting activities ensure a balanced number and position of COs, their identity and mechanism of action in mammals remain understudied. Previous work in yeast and Arabidopsis has shown that Zip2 and Shoc1 are ortholog proteins with an important role in promoting the formation of COs. Our work is the first study in mammals showing the *in vivo* and *in vitro* function of mouse and human SHOC1. We show that purified recombinant human SHOC1, an XPF/MUS81 family member, preferentially binds branched DNA molecules but apparently lacks *in vitro* endonuclease activity, despite its conserved ERCC4-(HhH)_2_ core structure. Cytological observations suggest that initial steps of recombination are normal in a majority of spermatocytes from SHOC1 hypomorphic mice. However, late stages of recombination appear abnormal, as chromosomal localization of MLH1 is reduced. In agreement, chiasma formation is reduced, and cells arrest at metaphase I with a few lagging chromosomes and subsequent apoptosis. This analysis of SHOC1-deficient mice and the selective localization of SHOC1 to a subset of recombination sites show that SHOC1 acts at key mid-stage steps of the CO formation process. The formation of chromosome axial elements and homologous pairing are apparently normal, but synapsis is altered with SYCP1 frequently failing to extend the full length of the chromosome axes. Finally, we describe that SHOC1 interacts with TEX11, another protein important for the formation of COs, connecting SHOC1 to chromosome axis and structure.

## Author summary

Meiosis is a specialized type of cell division in which haploid gametes are generated to counterbalance the doubling of the chromosome number occurring at fertilization. Proper chromosome segregation requires pairwise associations of the maternal and paternal homologous chromosomes. This is provided by chiasmata, which are generated as a result of homologous recombination-mediated repair of double-strand DNA breaks (DSBs). The association of meiotic chromosomes in pairs through chiasmata guarantees that each gamete receives only one copy of each chromosome. For this reason, errors in recombination are commonly linked to spontaneous abortions, aneuploid-based birth defects, and in some cases, infertility. We observed that the absence of SHOC1 results in deficient recombination and incomplete meiosis-specific modification of chromosome structure. Our findings thus indicate that the mouse SHOC1 protein is required for normal progression of important aspects of meiosis that act to ensure that the correct numbers of chromosomes are transferred to the next generation.

## Introduction

Cells in meiosis undergo two rounds of chromosome segregation after only one round of DNA replication. This highly regulated, specialized cell division generates haploid gametes containing a single copy of each chromosome. For maternal and paternal copies of each chromosome to segregate properly, the homologs must first pair with their correct partner and then become physically connected by chiasmata, the cytological representation of crossover (CO) products from homologous recombination (HR). The tether provided by COs allows the chromosome pair to orient correctly on the meiotic spindle [1]. Thus, mutations that alter recombination increase meiotic chromosome segregation errors and can result in aneuploid gametes.

In most organisms, meiotic recombination is initiated by chromosomal DNA cleavage by the SPO11 protein [2, 3]. The DNA ends at DSBs are resected to produce single-stranded DNA that binds two recombinases, RAD51 and DMC1. During strand invasion, these nucleoprotein filaments invade an intact homologous sequence [4, 5] to form single end heteroduplex invasion intermediates, which can be extended by DNA synthesis. Strand invasion intermediate processing may occur by one of two distinct pathways. The intermediates may dissociate with subsequent re-joining of the broken ends by synthesis-dependent strand annealing to generate non-crossovers (NCO). Alternatively, the initial strand invasion intermediate may become stabilized by 3′ to 5′ unwinding of duplex DNA that promotes synthesis-mediated extension, which induces more stable single-end invasions [6–8] and double Holliday junctions (dHJs) [7, 9]. These last two intermediates have been shown to occur in yeast only. Intermediates in the second pathway complete repair to generate COs [10]. Two distinct classes of COs are formed [11–13]. The majority of COs (90–95% in the mouse) are formed through multiple steps occurring throughout meiotic progression that require the ZMM group of proteins, which includes the SYCP1 protein from the transverse filaments of the synaptonemal complex [14], the ATP-dependent helicase Mer3/HFM1 [15] and the MSH4-MSH5 complex [16]. This class of COs (Class I) exhibits unique properties such as interference [17], which is defined as the non-random placement of crossovers, such that the formation of one crossover affects the likelihood of formation of a second crossover in an adjacent region. The second class of COs (Class II) involves structure specific endonucleases like MUS81 [18]. Although the regulation of crossing-over is critical in meiosis of all organisms, we still do not know the identity of several key activities and control mechanisms in mammals.

Zip2 is a yeast protein with an important role in meiosis. In the absence of Zip2, homologous chromosomes are properly paired (aligned) but not synapsed (juxtaposed and intimately bound by the synaptonemal complex). It has been proposed that Zip2 promotes initiation of chromosome synapsis by localizing at sites of interhomolog recombination [19]. Later work showed that in the absence of Zip2, single-end invasion intermediates form at wild type levels, but are maintained at peak levels for much longer than in wild type cells. Furthermore, double-Holliday junction patterns are also defective and yield a 40–50% reduction in the number of COs, which appear with substantial delay with respect to wild type. Defects in CO formation are even higher when specific hotspots are analyzed [20–22]. *zip2* mutations also prevent the formation of normal synaptonemal complex [22].

Later work on *Arabidopsis thaliana* revealed that the Shoc1 protein, a novel member of the ZMM group of proteins and an ortholog of Zip2, is required for class I CO formation [23]. Plants lacking Shoc1 protein show impaired meiosis but show no other developmental defects. Shoc1 mutant meiocytes resemble those from wild type plants at the leptotene and pachytene stages, with apparent normal homologous chromosome pairing and synapsis. However, cells at diakinesis show a mixture of univalents and bivalents, revealing reduced numbers of chiasmata, which causes an uneven distribution of chromosomes after anaphase I and unbalanced chromosome numbers in spores after the second meiotic division. Dmc1 focus numbers are not affected, but chiasmata frequencies in Shoc1 mutants are significantly lower than in wild type. Further, Arabidopsis *Shoc1* belongs to the same epistatic group as *Msh5*, which acts with Msh4 and Zip4 in the same pathway for CO formation.

A distinctive feature of plant Shoc1 proteins is a highly conserved region with an Ercc4-helix-hairpin-helix (HhH)_2_ core typically found in Xpf/Mus81 family members and suggestive of endonuclease and DNA binding activity. The (HhH)_2_ structure typically consists of two consecutive HhH motifs that are linked by a connector helix, and is often involved in non-sequence specific DNA binding. This core can be used to identify homologs in Fungi, Metazoa, Mycetozoa, and mammals via a reciprocal PSI-BLAST procedure [23]. Indeed, Arabidopsis Shoc1 has a high structural similarity to human (C9ORF84), mouse (AI481877) (SHOC1/ZIP2H), and *S*. *cerevisiae* (Zip2) proteins, which indicates these are probably orthologs. Xpf/Mus81 protein family members are conserved in eukaryotes and archaea, where they exist as either heterodimers or homodimers, respectively. Humans and mice have three endonucleases with the catalytic activity first known as XPF-ERCC1, MUS81-EME1, and MUS81-EME2. Vertebrates including humans have two additional XPF/MUS81 family members, FANCM and FAAP24, which form heterodimers with no detected endonuclease activity [24]. Within a heterodimer, the catalytic subunits share a characteristic core containing an ERCC4-like nuclease domain (GDX_n_ERKX_3_D) and a tandem (HhH)_2_ domain. This ERCC4-(HhH)_2_ arrangement also occurs in non-catalytic subunits, but they have highly diverged sequences. For example, the ERCC4 domain in human FANCM lacks the lysine side chain, which is consistent with observations that FANCM-FAAP24 has no endonuclease activity [25, 26]. This may reflect non-catalytic roles for these domains, such as binding ssDNA or targeting to defined DNA structures or sites of DNA damage [27, 28].

A minimum number of COs must be formed in a cell to connect pairs of homologous chromosomes and promote their proper segregation on the first meiotic spindle. Although pro-crossover activities play a central role in this process by ensuring that a subset of DSBs is repaired into COs, several of these proteins in mammals are unidentified, and for those we have recognized, their mechanisms of action remain poorly understood. While recent studies of plant Shoc1 suggest that the functionality of Shoc1 depends on the formation of a Shoc1-Ptd1 complex [29] similar to that described for Xpf-Ercc1, we still do not understand the function of SHOC1 orthologs in mammalian meiosis. Our studies integrate *in vitro* biochemical mechanisms of recombination mediated by SHOC1 and the meiotic phenotype of *Shoc1* deficient mice to provide a comprehensive functional and mechanistic understanding of the SHOC1 protein in mammalian meiosis.

## Results

### Kinetics of SHOC1 association to meiotic chromosomes

**Fig 1:**
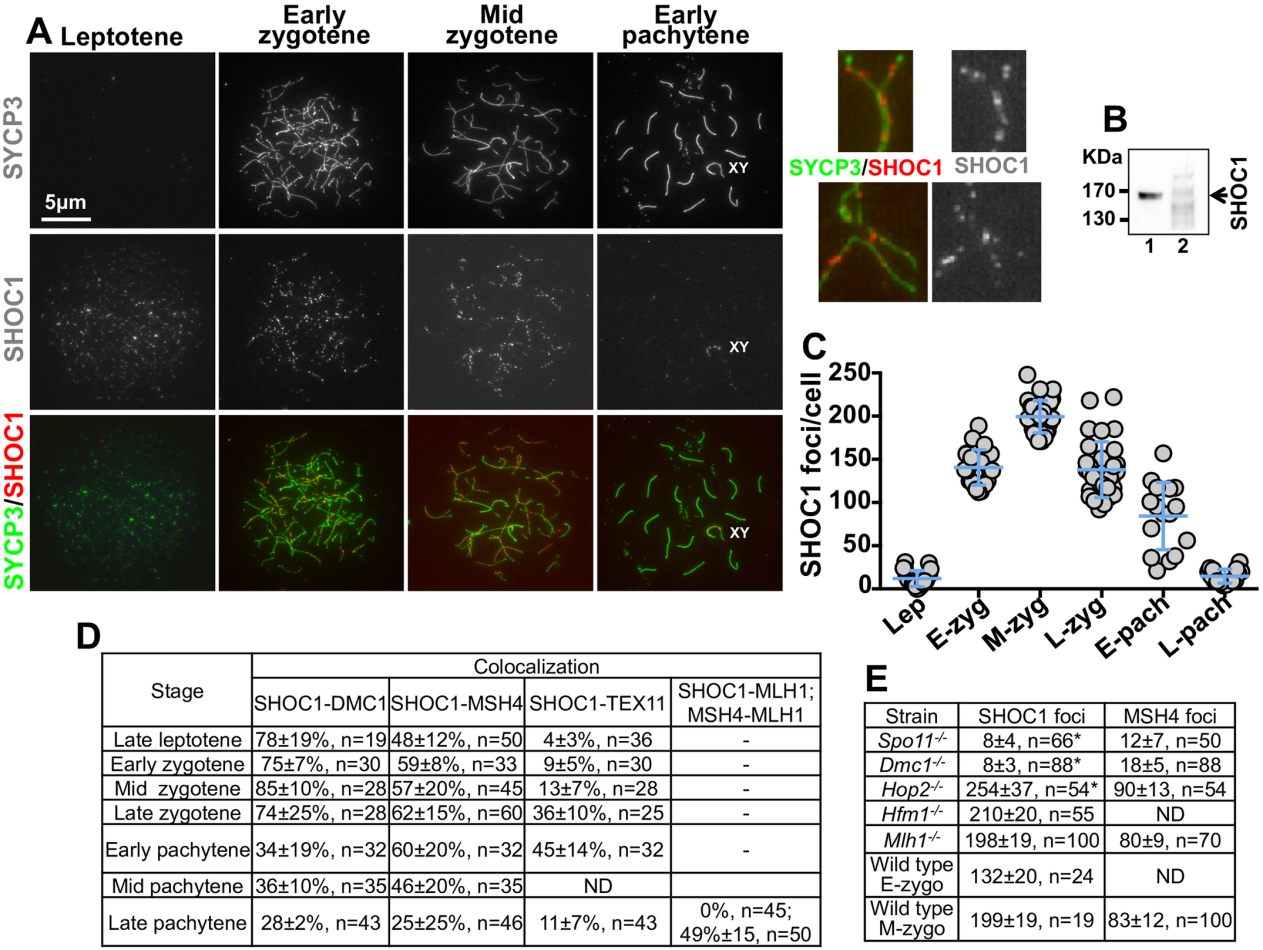
Kinetics of SHOC1 association to chromosomal recombination sites. (A) Wild type mouse spermatocytes at different stages of prophase I immunostained with anti-SYCP3 and anti-SHOC1 antibodies (insets on the upper right show higher magnification images of cells in zygotene). (B) SHOC1 antibodies detect a unique band in a testis extract Western blot (1) and pre-incubation of the SHOC1 antibody with the protein fragment used to generate this antibody (540–1250 amino acids) prevents detection of the SHOC1 protein in testis extracts (2). (C) Quantification of SHOC1 foci shown in A (mean ± SD). Leptotene cells (Lep) 12±9, n = 21; Early Zygotene cells (E-zyg) 140±20, n = 24; Mid zygotene cells (M-zyg) 199±19, n = 37; Late zygotene cells (L-zyg) 138±32, n = 32; Early pachytene cells (E-pach) 84±39, n = 16; Late pachytene cells (L-pach) 15±8, n = 16. Spermatocytes from 3 wild type 13 day-old mice were analyzed. (D) Co-localization of SHOC1 with DMC1, MSH4, TEX11, and MLH1 (expressed as the percentage of SHOC1 chromosomes positive for the second marker). Spreads were obtained from ≥3 wild type 13 day-old mice. For MLH1 experiments we used wild type 45 day-old mice. (E) Quantification of SHOC1 and MSH4 foci in wild type meiocytes and spermatocytes knocked out for *Spo11*, *Dmc1*, *Hop2*, *Hfm1*, and *Mlh1* genes. Asterisks represent values that are significantly different compared to wild type (P<0.0001). We compared wild type spermatocytes at early zygotene stage (E-zygo) with *Spo11*^*-/-*^, *Dmc1*^*-/-*^, and *Hop2*^*-/-*^ spermatocytes; and wild type spermatocytes at mid zygotene stage (M-zygo) with *Hfm1*^*-/-*^ and *Mlh1*^*-/-*^ spermatocytes. Spreads for these experiments were obtained from ≥2 mice for each mutant.

We first used immunocytochemistry to assess the kinetics of SHOC1 association and dissociation at recombination sites in mouse meiotic chromosomes. We used rabbit polyclonal antibodies specific for mouse SHOC1 (Fig 1A and 1B). SHOC1 antibodies recognize only one band at approximately the expected molecular weight in Western blots of wild type testis extract. Preincubation of SHOC1 antibodies with a SHOC1 protein fragment used to produce these antibodies abolishes reactivity against SHOC1 in Western blots (Fig 1B). Indirect immunofluorescent localization of SHOC1 in wild type spermatocyte chromosome spreads showed that a maximum number of foci can be detected at mid zygotene stage (199 ± 19 foci/cell, mean ± SD, n = 37) (Fig 1A and 1C). No signal was detected when we used this antibody to immunostain *Shoc1*^*hyp/hyp*^ spermatocyte spreads (see description of this mutant below and S1 Fig). This may reflect the lack of sensitivity of SHOC1 antibodies to reduced amount of SHOC1 in the mutant mice (see below) or inability of the remaining SHOC1 to accumulate at recombination sites. Our results suggest a role of SHOC1 in the intermediate stages of recombination.

### SHOC1 associates with mid-stage meiotic recombination intermediates

We propose a model in which SHOC1 loading at meiotic chromosomes coincides with the time recombination intermediates stabilize pairwise associations of homologous chromosomes. To test this model, we evaluated the percent co-localization of SHOC1 foci with DMC1 (which catalyzes strand invasion), MSH4 (which acts at intermediate stages of recombination), TEX11 (essential for meiotic recombination intermediate metabolism) [30, 31] and MLH1 (presumably involved in resolution of DNA branched structures and a marker of CO sites) (Fig 1D and S2 Fig). We observed strong co-localization between SHOC1 and DMC1 (maximum at mid zygotene spermatocytes, 85±10% SHOC1 overlapping with DMC1, n = 28; 99±2% DMC1 overlapping with SHOC1, n = 30). At this stage we observed 177±16 DMC1 foci per cell (n = 55). We note that the relatively high percentage of co-localizing SHOC1 and DMC1 foci at late pachytene spermatocytes (28±2%, n = 43), where DMC1 is nearly absent at the chromosome axis, mostly correspond to foci located on X-Y chromosomes.

**Fig 2:**
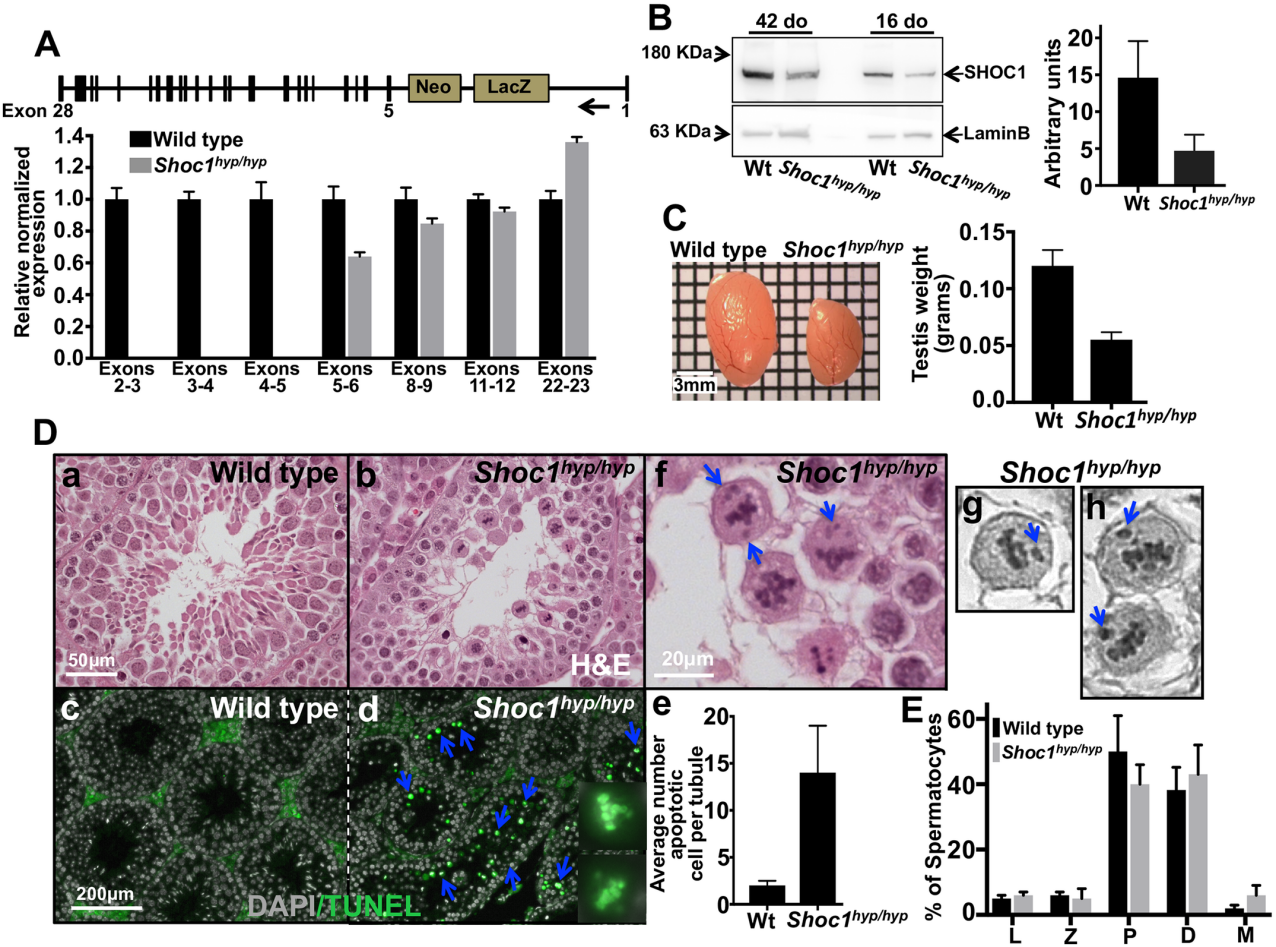
SHOC1 deficient mice show profound defects in gametogenesis. (A) *Shoc1* gene targeting design and expression of *Shoc1* measured by RT-PCR in mutant mice. A total of three 16 day-old mice of each genotype were analyzed. (B) Sixteen and 42 day old (do) *Shoc1*^*hyp/hyp*^ mice show reduced amounts of SHOC1 protein compared to wild type mice. Quantification of protein levels was measured in five total mice for each genotype, two at 16 days and three at 42 days of age. (C) *Shoc1*^*hyp/hyp*^ mice have reduced testis size and weight compared to wild type mice. (D) Meiosis is arrested at the end of prophase I in *Shoc1*^*hyp/hyp*^ spermatocytes (a, wild type and b, *Shoc1*^*hyp/hyp*^ mice) with an increased number of apoptotic cells (c and d, blue arrows). Higher magnification of two apoptotic cells is also shown. Magnification bar in a corresponds to images in a and b and magnification bar in c corresponds to images in c and d. Apoptotic cells are quantified in e (n = 435 day-old mice/genotype). *Shoc1*^*hyp/hyp*^ metaphase I spermatocytes show a high number of lagging chromosomes (f, higher magnification in g and h) (see Fig 3D and text for details and quantification). (E) Stages of meiosis of spermatocytes from wild type and *Shoc1*^*hyp/hyp*^ mice. L, leptotene cells; Z, zygotene cells; P, pachytene cells; D, diplotene cells; M, metaphase I cells. Random spermatocyte spreads were scored from 60 day-old mice (n = 3).

We also observed substantial co-localization between SHOC1 and MSH4 (maximum at late zygotene cells, 62±15%, n = 60) and SHOC1 and TEX11 at later meiotic stages (maximum at early pachytene cells, 45±14%, n = 32) but detected no significant co-localization between residual SHOC1 foci present at late pachytene spermatocytes and MLH1 foci (0%, n = 45). In contrast, MSH4-MLH1 showed a maximum of 49% of foci co-localizing at late pachytene spermatocytes (Fig 2D). These data suggest that SHOC1 may be necessary at intermediate stages of prophase I, where it could stabilize intermediates of the recombination pathway (see below) and promote stable homologous chromosome interactions, but may be absent from later recombination structures marked by MLH1.

### SHOC1 localization at meiotic chromosomes requires DSBs and recombination intermediates catalyzed by DMC1

To more precisely pinpoint the stage at which SHOC1 is involved in the recombination process we analyzed its localization to chromosomes in mutant mice that block progression of the HR pathway at specific stages (Fig 1E). We monitored SHOC1 localization at the early zygotene-like stage (based on SYCP3 immunostaining and SYCP3/SYCP1 co-localization) in *Spo11*^-/-^ spermatocytes, which have no DSBs [2, 3], *Dmc1*^*-/-*^ spermatocytes which are defective in strand invasion [32], *Hop2*^*-/-*^ spermatocytes, in which DMC1 interacts with resected DSBs but progression of strand invasion is impaired [33], spermatocytes deficient for HFM1, in which initial strand invasion intermediates are stabilized [15] and *Mlh1*^*-/-*^ spermatocytes, which are blocked in the final stages of CO formation [34]. We scored cells for the average number and size of axial-lateral element fragments per cell and monitored MSH4 foci as a reference since this protein is known to stabilize intermediate stage recombination events. Our results show that the number of SHOC1 foci was substantially reduced in both *Spo11*^*-/-*^ (8±4 foci/cell, n = 66) and *Dmc1*^*-/-*^ (8±3 foci/cell, n = 88) spermatocytes compared to wild type spermatocytes (132±20 foci/cell, n = 24, P<0.0001). In contrast, the number of SHOC1 foci was significantly increased in *Hop2*^*-/-*^ spermatocytes (254±37 foci/cell, n = 54) and no change in the number of SHOC1 foci was observed in *Hfm1*^*-/-*^ and *Mlh1*^*-/-*^ spermatocytes compared to mid-zygotene wild type cells. The increase of SHOC1 foci in *Hop2*^*-/-*^ spermatocytes *was* unexpected because HOP2 has been proposed to work in cooperation with DMC1 catalyzing formation of strand invasion intermediates of recombination [33, 35–37]. It is possible that in *Hop2*^*-/-*^ spermatocytes, SHOC1 is able to interact with recombination intermediates (i.e. strand invasion) or their associated proteins, but progression of these intermediates to later stages is impaired.

In summary, a number of results suggest that mid-stage DNA recombination intermediate structures, and possibly their associated proteins, are required for optimal SHOC1 loading on recombination sites. These are: 1) kinetics of SHOC1 association with meiotic chromosomes; 2) the requirement of DMC1 for normal SHOC1 foci numbers; 3) the high number of co-localizing SHOC1/DMC1 foci, and 4) the increased number of SHOC1 foci in *Hop2*^*-/-*^ spermatocytes, which accumulate DMC1/RAD51-single-stranded DNA nucleoprotein filaments [33]. The observations that DMC1 is apparently required for normal levels of SHOC1 foci, and that RAD51 foci numbers in spermatocytes are not affected by changes in the wild type level of SHOC1 (see below), support for a model in which SHOC1 acts downstream of DMC1.

### Normal progression of meiosis requires *Shoc1*

Although deletion of *Shoc1* in Arabidopsis results in meiotic defects, the requirement for SHOC1 in mammalian meiosis is still not understood. Thus, we undertook a study of the role of SHOC1 in mammalian meiosis. To generate SHOC1 deficient mice we designed a gene trap vector that replaced coding exons 2–20 of *Shoc1* with a Neo-PGK-gb2 cassette (S3A Fig, see Methods). We confirmed the correct insertion of Neo-PGK-gb2 by PCR followed by sequencing (S4 Fig). *Shoc1* heterozygous mice were fertile, did not display any apparent tissue anomalies and showed a normal life span. However, when heterozygous mice were mated, the genotyping of 76 offspring from 15 different litters yielded no pups homozygous for the mutant *Shoc1* allele (S3B Fig). We confirmed these results by analyzing the genotypes of the yolk sacs and embryos from 9.5 dpc embryos. Again, no homozygous mutant mice were detected (S3C Fig). Thus, we were not able to obtain male or female mice carrying the above-mentioned mutation for meiotic analysis. This was unexpected, as *Shoc1* is mostly expressed in testis of adult mice (S3D Fig). It is possible that the absence of homozygous mutant mice may be caused by embryonic lethality caused by the absence of SHOC1, consistent with results showing that *Shoc1* is expressed during murine embryonic development (S3E Fig). However, other scenarios are possible such as a linked mutation.

In a separate attempt to generate *Shoc1* knockout mice, we created heterozygous mice in which only coding exons 2–4 were replaced with a neomycin-resistance cassette, which should introduce a frame shift mutation (Fig 2A and S3F Fig). We obtained wild type, heterozygous, and homozygous mutant mice at the expected mendelian ratios (1:2:1). RT-PCR analysis of transcript levels for exons across the gene from testes of homozygous targeted mice showed that expression of *Shoc1* is altered but not abolished by the gene trap insertion. While expression corresponding to exons 1–4 was undetectable (transcription start site is predicted at exon 2), we observed significant expression of downstream exons (Fig 2A), which suggests that an alternate transcription start site present in exon 5 is activated after deletion of exons 2–4. Although we observed some expression of RNA transcript, comparative analysis of testis from wild type and genetically modified mice by Western blot revealed that the amount of SHOC1 (calculated wild type SHOC1 MW: 168.06 KDa) is reduced in SHOC1 defective mice (Fig 2B). Elimination of exons 2–4 (coding 84 amino acids) may result in a truncated version of SHOC1 with a calculated MW of 158 KDa. This short version of SHOC1 cannot be distinguished from wild type in Western blots, likely due to the lack of resolution of the SDS-PAGE gels we used. Thus, we generated hypomorph mice (*Shoc1*^*hyp*^) expressing reduced levels of a truncated version of SHOC1. This partial loss of SHOC1 allows embryonic viability and development throughout adulthood.

To test whether *Shoc1*^*hyp/hyp*^ mice exhibit meiotic defects, we first carried out testis tissue and germ cell analysis. Testes of *Shoc1*^*hyp/hyp*^ mice (n = 10) are significantly smaller (0.055g±0.0067 vs. 0.12g±0.014; mean ± SD, P = 0.0006, t test, Fig 2C) compared to wild type testes (n = 8), a phenotype commonly seen in mice with meiotic defects [15, 16, 34]. Tissue analysis indicated that *Shoc1*^*hyp/hyp*^ males undergo incomplete testis development with enlargement of interstitial cells and lack of spermatozoa. The number of seminiferous tubules was not reduced, but their size (calculated as the average diameter per tubule) was decreased by an average of 23±13% (307±27 μm for wild type and 238±41 μm for *Shoc1*^*hyp/hyp*^*)* (n = 20 wild type and n = 26 *Shoc1*^*hyp/hyp*^ seminiferous tubules analyzed from three different mice of each genotype) compared to wild type mice (S1 Table). Primary spermatocytes represented the most advanced spermatogenic cells detected in the *Shoc1*^*hyp/hyp*^ mice, indicating that spermatogenesis was blocked at metaphase of meiosis I (Fig 2Da–2Dh).

The *Shoc1*^*hyp/hyp*^ mutant testes showed signs of extensive apoptosis in seminiferous tubules (average number of apoptotic cells per positive tubule ± standard deviation, 13.7±4.6, n = 50 seminiferous tubules obtained from tissue sections of four different mice, Fig 2Dc–2De) compared to wild type (1.98±0.58, n = 50 seminiferous tubules scored from two mice, P = 0.0001). Approximately 98% of TUNEL positive cells (90 seminiferous tubules scored) were at the metaphase I stage.

We analyzed spermatocyte progress through meiotic prophase I in detail by scoring individual stages of asynchronous populations of wild type and *Shoc1*^*hyp/hyp*^ spermatocytes from adult mice (Fig 2E). We observed increases in the percentages of diplotene (43±9% vs. 38±7%; mean ± SD; P≤0.0001, two tailed t test) and metaphase I spermatocytes (6±3% vs. 2±1%; P≤0.0005) and a decrease in pachytene spermatocytes (40±6% vs. 50±11%; P≤0.0001) in *Shoc1*^*hyp/hyp*^ mice (n = 1,198 cells from 3 mice) relative to wild type mice (n = 1,360 cells from 3 mice).

We also analyzed hematoxylin-eosin histological sections of 35-day-old wild type and *Shoc1*^*hyp/hyp*^ ovaries. Compared with wild type mice, no significantly changes in ovary size or number of follicles and corpora lutea were observed in *Shoc1*^*hyp/hyp*^ mice (S5 Fig). This is similar to the phenotype observed for *Mlh1*^*-/-*^ mutants ([34]. Matings of homozygous *Shoc1*^*hyp/hyp*^ female mice with wild type males produced normal number and sizes of litters compared with matings of wild type mice (S2 Table). Together, the results suggest no effect of the *Shoc1*^*hyp*^ mutation in mouse female gonad development. Although the results suggest sexual dimorphism with respect to the deletion of *Shoc1*, it is also possible that the lack of a phenotype in *Shoc1*^*hyp/hyp*^ ovaries is caused by the incomplete ablation of SHOC1.

**Fig 3:**
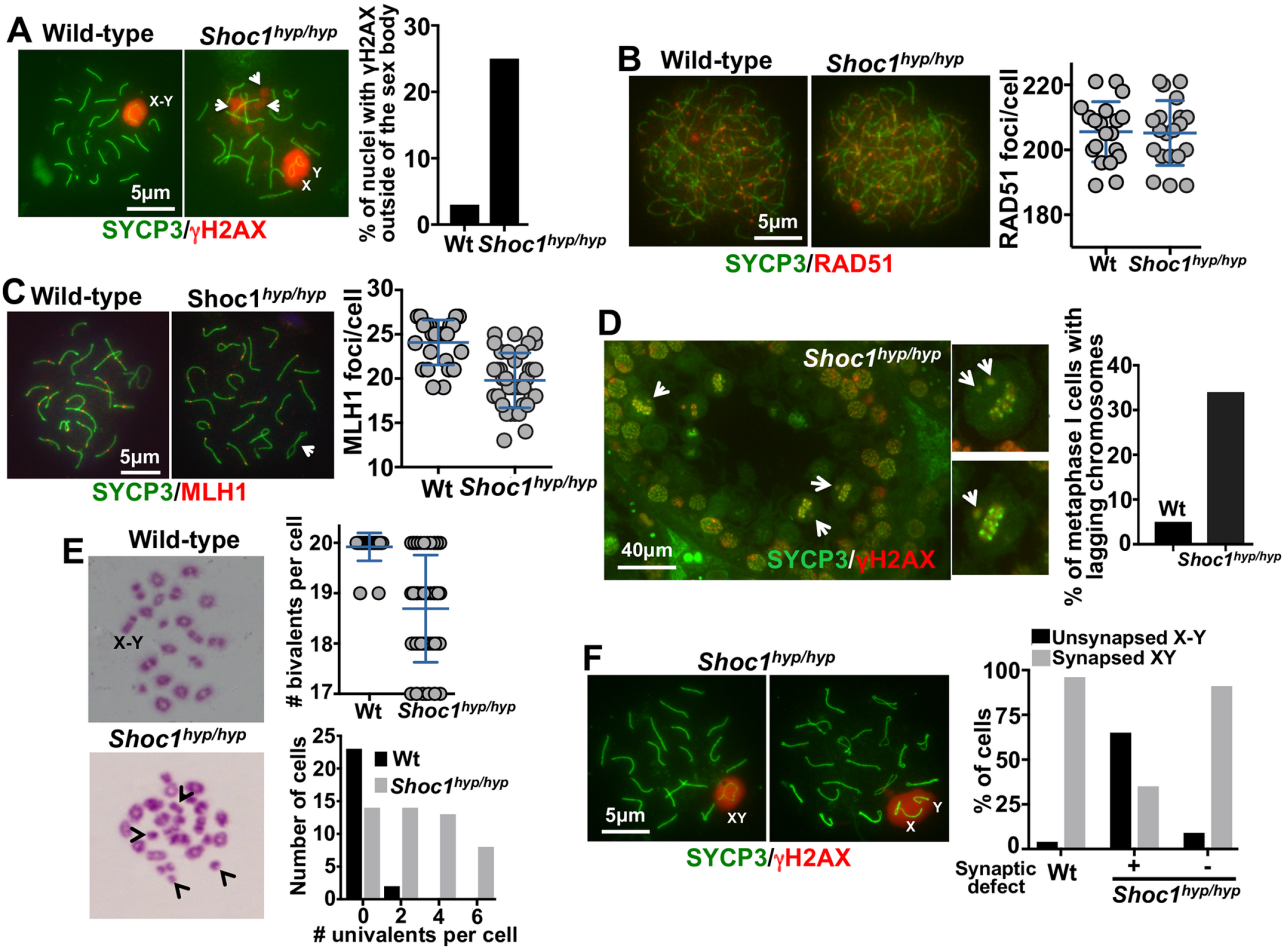
*Shoc1*^*hyp/hyp*^ spermatocytes show defects in DSB repair and reduced crossover formation. (A) Representative chromosome spreads of wild type and *Shoc1*^*hyp/hyp*^ spermatocytes immunostained with SYCP3 and γH2AX antibodies. Quantification of random spermatocytes showing an additional γH2AX signal outside the sex body is also shown (n = ≥3 45 day-old mice/genotype). (B) Representative chromosome spreads of wild type and *Shoc1*^*hyp/hyp*^ spermatocytes immunostained with SYCP3 and RAD51 antibodies. Quantification of RAD51 foci/cell in random wild type and *Shoc1*^*hyp/hyp*^ spermatocytes at zygotene stage (n = ≥3 13 day-old mice/genotype, mean ± SD). (C) Representative chromosome spreads of wild type and *Shoc1*^*hyp/hyp*^ spermatocytes immunostained with SYCP3 and MLH1 antibodies. Arrow indicates a synaptic deficient chromosome with no MLH1 foci. Quantification of MLH1 foci/cell for random wild type and *Shoc1*^*hyp/hyp*^ spermatocytes also shown (n = ≥3 45 day-old mice/genotype, mean ± SD). (D) SYCP3 and γH2AX staining of a representative *Shoc1*^*hyp/hyp*^ testis section. Quantification of number of spermatocytes with at least one laggard chromosome/metaphase I wild type and *Shoc1*^*hyp/hyp*^ spermatocyte also shown (n = ≥3 45 days old mice/genotype). (E) Representative metaphase spreads of wild type and *Shoc1*^*hyp/hyp*^ spermatocytes. Note the increased number of univalents (arrows) in *Shoc1*^*hyp/hyp*^ cells. X and Y indicate the sex chromosomes. Quantification (mean ± SD) of metaphase bivalents per cell and number of cells versus number of univalents per cells in wild type and mutant mice also shown (n = ≥3 45 day-old mice/genotype, mean ± SD). (F) Representative pachytene wild type and *Shoc1*^*hyp/hyp*^ spermatocytes immunostained for SYCP3 and γH2AX showing synapsed or unsynapsed X and Y chromosomes within the sex body. For quantitation, cells from *Shoc1*^*hyp/hyp*^ mice were divided in two categories according to the severity of synaptic defects. Spermatocytes were from two wild type and three *Shoc1*^*hyp/hyp*^ 45 day-old mice.

In summary, tissue analysis suggests that spermatogenesis progresses normally in *Shoc1*^*hyp/hyp*^ male mice until the end of prophase I when spermatocytes arrest at metaphase I, which may indicate that prophase I checkpoints are not triggered. Chromosomes in *Shoc1*^*hyp/hyp*^ spermatocytes appear normally condensed at metaphase, but a few chromosomes are off the metaphase plate in contrast to chromosomes in wild-type spermatocytes (Fig 2Df–2Dh). A high number of metaphase I spermatocytes with lagging chromosomes in *Shoc1*^*hyp/hyp*^ mice suggests defects in chiasma formation (see text below and Fig 3D for a detailed quantification).

### Cytological markers suggest defective late stages of recombination in *Shoc1*^*hyp/hyp*^ spermatocytes

We then evaluated the progression of recombination in mouse *Shoc1*^*hyp/hyp*^ spermatocytes that appeared to be in a pachytene-like stage based on the association of the X and Y chromosomes. We assessed DSB repair by immunostaining chromosomes for γH2AX[15], which appears on chromatin near DSBs and disappears after repair. Although immunostaining with γH2AX marks only the sex body for most wild-type pachytene cells (3.2% γH2AX staining outside the sex body, n = 95, Fig 3A), a fraction (25%) of *Shoc1*^*hyp/hyp*^ spermatocytes showed persistent γH2AX staining not only in the sex body, but also in one or several patches along autosomes (n = 120, Fig 3A). The persistence of γH2AX into the pachytene stage is a hallmark of a failure or delay in the repair of meiotic DSBs [38].

After the resection of DNA ends, RAD51 binds to single-stranded DNA and catalyzes invasion of an intact homologous sequence [4, 5] to form cytologically detectable intermediates that likely mark single-end invasion sites [39]. To test whether these recombination stages are affected in *Shoc1*^*hyp/hyp*^ spermatocytes, we determined the number of RAD51 foci on meiotic chromosomes from wild type and *Shoc1*^*hyp/hyp*^ zygotene spermatocytes. There were no significant differences in the number of RAD51 foci/cell in wild type compared to *Shoc1*^*hyp/hyp*^ spermatocytes (205.6±9.3 vs. 205.2±10 foci/cell, n = 20, p = 0.9, unpaired t test, Fig 3B). We concluded that recombination stages involving RAD51 are not altered in *Shoc1*^*hyp/hyp*^ cells compared to wild type.

As CO formation in mice requires the MLH1 mismatch repair protein, the subset of recombination intermediates that mature into COs begins to be marked by MLH1 accumulation in early to mid pachytene spermatocytes [40]. To test whether recombination intermediates are processed to this stage in *Shoc1*^*hyp/hyp*^ spermatocytes, we immunostained for MLH1. *Shoc1*^*hyp/hyp*^ spermatocytes showed a significant reduction in the number of MLH1 foci/cell compared to wild type spermatocytes (19.8±0.5 MLH1 foci/cell, n = 38 versus 24.1±0.5 MLH1 foci/cell, n = 25, unpaired t test p<0.0001, Fig 3C). This reduced number of MLH1 foci suggests a role for SHOC1 in the class I crossover pathway. We note that this moderate phenotype is similar to that observed in mice deficient for the TEX11/ZYP4h protein [30, 31], which is a SHOC1 interaction partner (see below). In an alternative interpretation, residual SHOC1 present in the *Shoc1*^*hyp/hyp*^ spermatocytes may be able to facilitate loading of some MLH1 foci, but not to wild type levels.

### Chiasma frequency is reduced in *Shoc1*^*hyp/hyp*^ spermatocytes

To confirm that *Shoc1*^*hyp/hyp*^ deficient mice have reduced CO formation, we analyzed the frequency of *Shoc1*^*hyp/hyp*^ spermatocytes with lagging chromosomes at metaphase I. Achiasmate chromosomes fail to associate in the metaphase I plate and are often seen as lagging chromosomes (Fig 2D). We observed an elevated number of *Shoc1*^*hyp/hyp*^ spermatocytes at metaphase I with at least one lagging chromosome compared to wild type spermatocytes (5%, n = 60 wild type cells versus 34%, n = 100 *Shoc1*^*hyp/hyp*^ cells, Fig 3D). We note few lagging chromosomes (1.5±0.7/cell, mean ± SD, n = 100) in *Shoc1*^*hyp/hyp*^ spermatocytes, indicating that only some homologous chromosome pairs fail to form chiasmata.

Chiasmata join homologs to make stable bivalent chromosomes; when chiasma numbers are reduced, the numbers of bivalents are reduced and univalents are observed. So, we scored the number of bivalent chromosomes per cell in air-dried chromosome spreads and found significantly fewer bivalents/cell in *Shoc1*^*hyp/hyp*^ spermatocytes compared to wild type spermatocytes (18.7±0.15, n = 49 versus 19.9±0.05, n = 25, unpaired t test, p<0.0001, Fig 3E). Together with the reduction in MLH1 foci (Fig 3C), and an increased number of lagging chromosomes (Fig 3D), the reduction in the number of bivalents that we observed in *Shoc1*^*hyp/hyp*^ spermatocytes suggests that normal CO formation requires wild type levels of SHOC1.

In early pachytene spermatocytes, X and Y chromosome pairs normally form a short stretch of synaptonemal complex encompassing the small region of homology in the pseudo-autosomal region (PAR). Synapsis at PAR is sensitive to reduced rates of recombination and perturbations in synapsis, and thus can be used to detect defects in these processes. Although the sex body appeared to form normally in all pachytene and diplotene *Shoc1*^*hyp/hyp*^ spermatocytes, the X and Y chromosome PAR regions were unsynapsed and separated in 65% of *Shoc1*^*hyp/hyp*^ cells with obvious synaptic defects (n = 52) and in 9% of *Shoc1*^*hyp/hyp*^ cells with no apparent synaptic defects (n = 32) versus only 4% of wild-type cells (n = 50, P≤0.001, t test comparing wild type versus *Shoc1*^*hyp/hyp*^ cells with obvious synaptic defects, Fig 3F). We conclude that SHOC1 is required for normal levels of XY synapsis but not for sex body formation.

### Homologous chromosomes pair in *Shoc1*^*hyp/hyp*^ spermatocytes but show defective synapsis

**Fig 4:**
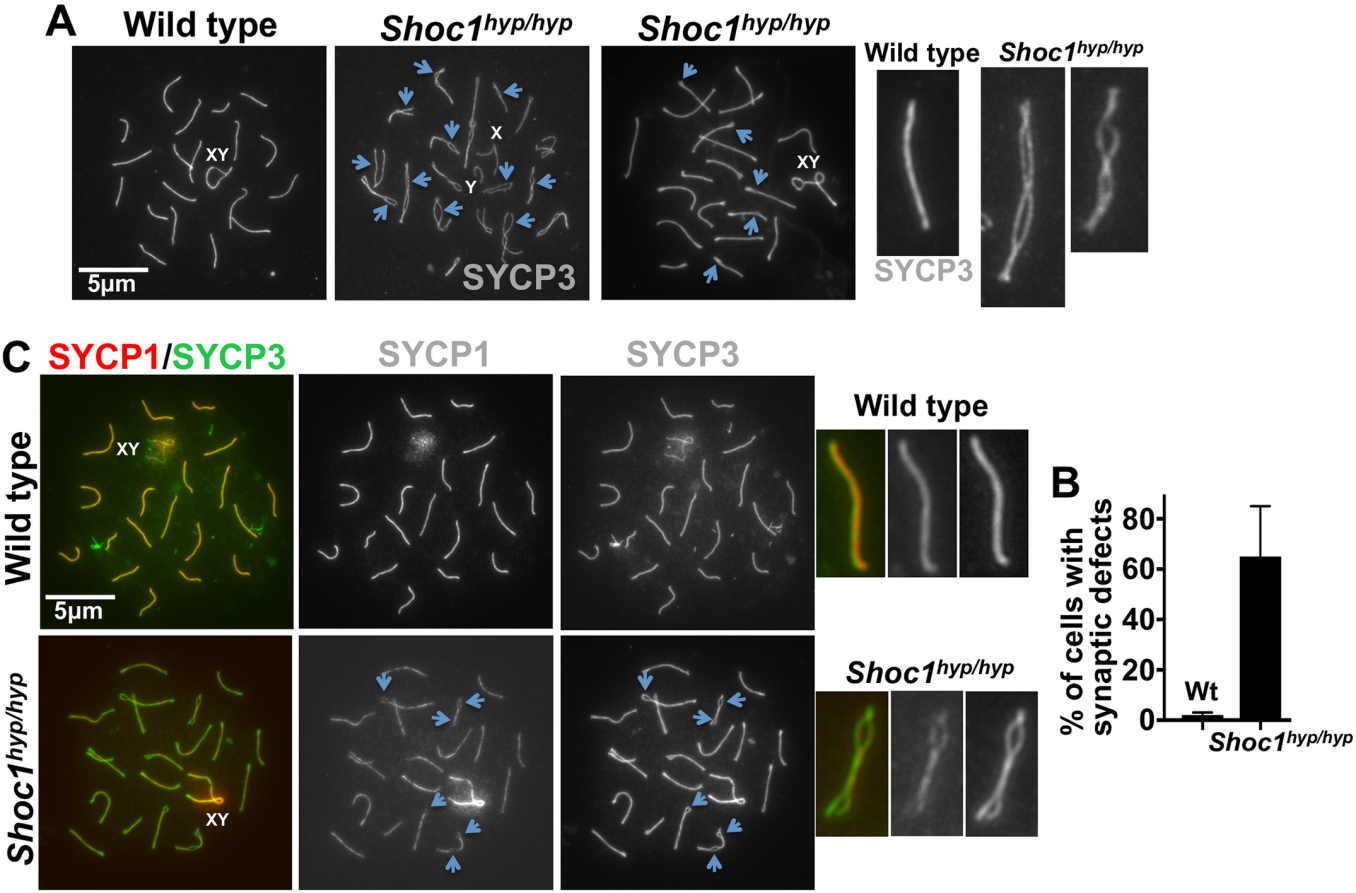
Synaptic defects in *Shoc1*^*hyp/hyp*^ spermatocytes. (A) SYCP3 immunostaining of wild type and *Shoc1*^*hyp/hyp*^ spermatocytes reveals that SHOC1 deficient spermatocytes undergo normal homologous chromosome pairing but have defective synapsis. Arrows mark sites of synaptic defects. X and Y represent the sex chromosomes. (B) Quantification of synaptic defects in wild type and *Shoc1*^*hyp/hyp*^ mice. (n = ≥3 45 day-old mice/genotype. (C) Representative wild type and *Shoc1*^*hyp/hyp*^ spermatocytes immunostained with SYCP1 and SYCP3 antibodies. Arrows mark areas showing a lack of synapsis.

Defects in meiotic recombination often result in defective homologous chromosome associations. We tested the requirement for SHOC1 in synapsis by immunolocalizing the synaptonemal complex proteins SYCP3 of the axial/lateral element and SYCP1 of the transverse filament in spermatocyte chromosome spreads. We found autosomal axes of similar lengths in co-aligned pairs of homologous chromosome from wild type (less than 1% of each other) and *Shoc1*^*hyp/hyp*^ spermatocytes (less than 5% of each other, S3 Table). Our measurements included the lengths of both juxtaposed axes (end to end SYCP3 signal) of 200 chromosomes with obvious synaptic defects from 20 *Shoc1*^*hyp/hyp*^ cells. Our results indicate that development of the axial element of the synaptonemal complex and homologous chromosome pairing are normal in all *Shoc1*^*hyp/hyp*^ spermatocytes (Fig 4A). In addition, the absence of splitting of the axes indicated that sister chromatid cohesion is not affected in *Shoc1*^*hyp/hyp*^ mice (Fig 4A).

Although spermatocytes progress through meiotic prophase I in *Shoc1*^*hyp/hyp*^ mice (Fig 2E), pachytene spermatocytes show synaptic defects (Fig 4A–4C). We analyzed the numbers and types of synaptic anomalies in pachytene cells (spreads were scored as in this stage if at least 95% of the axial/lateral elements (SYCP3 signal) of homologous chromosomes showed close co-alignment (synapsis), and sex chromosome axes showed the typical early pachytene configuration [41]). At least one abnormal synaptic conformation was found in approximately 65% of all scored *Shoc1*^*hyp/hyp*^ pachytene-like spermatocytes (n = 120) but approximately 2.5% of wild-type control spermatocytes (n = 40) (Fig 4B). *Shoc1*^*hyp/hyp*^ bivalents often had one end or an interstitial zone unsynapsed (Fig 4A and 4C). We confirmed synaptic defects using SYCP1 immunostaining, which revealed that this central region component of the synaptonemal complex was weakly associated with the SYCP3 axes at regions of chromosome separation (Fig 4C). Thus, although chromosome homologous recognition and pairing appears normal in *Shoc1*^*hyp/hyp*^ spermatocytes, synapsis is frequently defective.

### SHOC1 preferentially binds to single-stranded DNA and DNA branched structures

**Fig 5:**
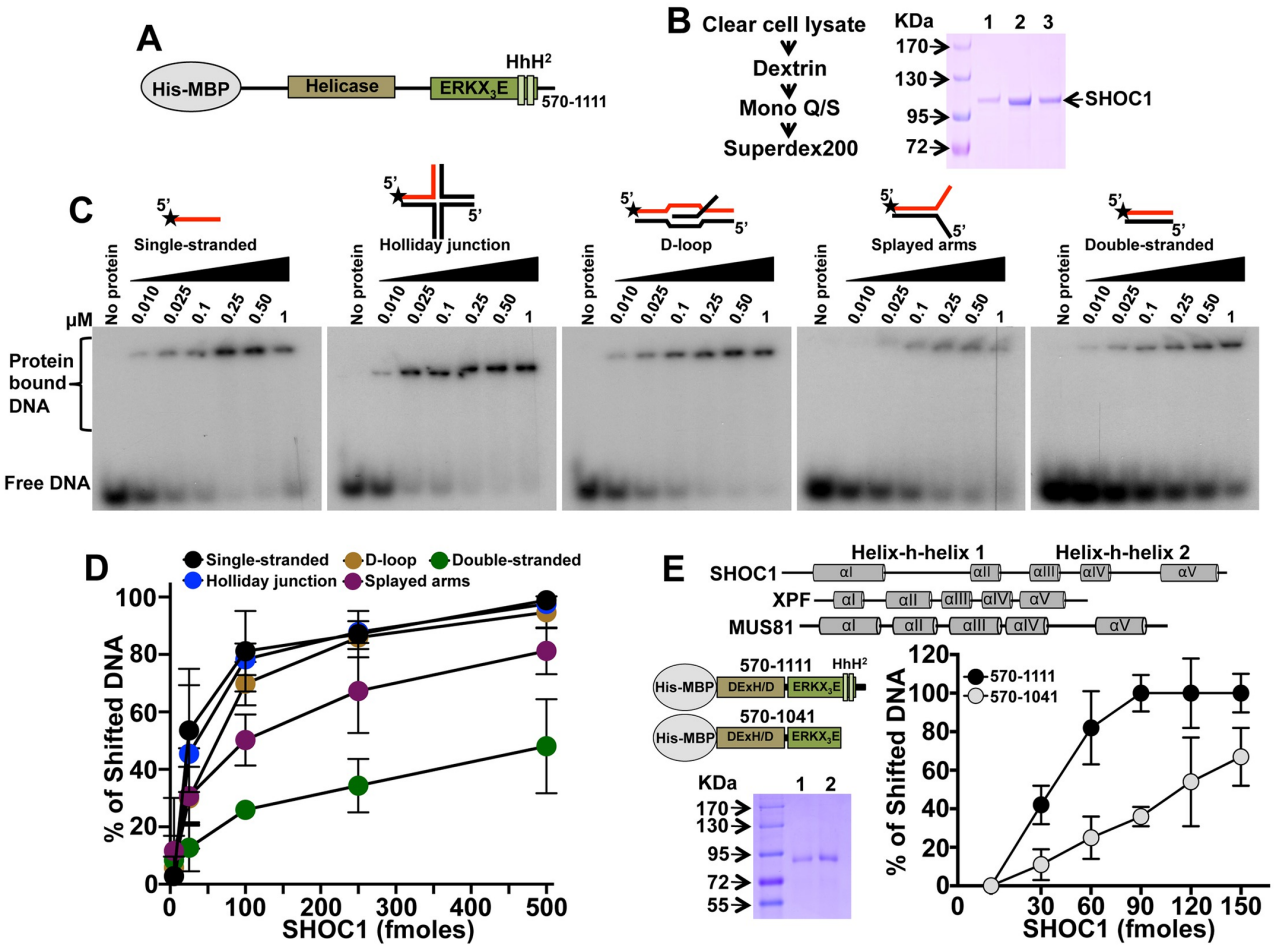
Shoc1 DNA binding specificity. (A) Schematic showing conserved motifs within human SHOC1^570-1111^. (B) Shoc1 purification protocol and Coomassie stained SDS-PAGE showing purified SHOC1^570-1111^. SHOC1^570-1111^ was purified to near homogeneity after Dextrin (1), Mono Q/S (2), and Superdex 200 (3) chromatography. (C) Representative native PAGE gel shift assays showing SHOC1^570-1111^ binding to distinct DNA substrates. (D) Quantitation of Shoc1 binding to DNA substrates shown in C (n = 2–3, mean ± SD). (E) Deletion of the (HhH)_2_ domain reduces human SHOC1 binding to single-stranded DNA. Schematic diagram of the SHOC1^570-1041^ mutant and structural characteristics of (HhH)_2_ domains of human XPF, SHOC1 and MUS81 are also shown. Coomassie stained SDS-PAGE showing purified SHOC1^570-1041^ is also shown. Mono Q/S (1), and Superdex 200 (2) chromatography.

Using the amino acid sequence of SHOC1 and 3D structural prediction (HHpred) algorithms [42], we found that mouse (AI481877) and human SHOC1 (C9orf84) show an ERCC4 nuclease-(HhH)_2_ core structure similar to that in the XPF/RAD1/MUS81 family of nucleases, which is in agreement with previous work describing the Arabidopsis orthologs [23]. Human SHOC1 contains a putative nuclease motif with sequence GEX_22_ERKX_3_E followed by two well-defined helix-hairpin-helix (HhH)_2_ domains similar to SHOC1 homologs from other closely related mammals (Fig 5A and S6A Fig). Although these structural parallels may indicate that SHOC1 exhibits characteristic biochemical activities of the XPF/MUS81 family of nucleases, we note that the endonuclease sequence diverges from the canonical GDX_n_ERKX_3_D [24].

To determine SHOC1 *in vitro* biochemical activities, we expressed and purified a truncated form of the protein, SHOC1^570-1111^, from insect cells. We adopted this strategy because we were unable to generate soluble full-length SHOC1 using multiple expression systems (E. coli, yeast, and insect cells), possibly due to a disordered C-terminal tail region. In the human SHOC1 protein sequence, the (HhH)_2_ domain is linked to a C-terminal tail region that appears naturally disordered (S6B Fig). We purified this fragment of human SHOC1 using a multistep protocol, including dextrin and mono Q/S affinity chromatography and gel filtration (Fig 5A and 5B). This SHOC1 fragment contains all predicted XPF functional domains, is active in biochemical assays (below), and is apparently monomeric (evaluated by chemical crosslink followed by SDS-PAGE gel electrophoresis and Superdex 200 gel filtration, S7A and S7B Fig).

As the presence of a ERCC4-(HhH)_2_ core structure is indicative of protein DNA binding, we used a DNA gel shift assay to identify preferred DNA substrates of SHOC1^570-1111^ (Fig 5C and 5D). We generated and purified different DNA branched structures (splayed arms, D-loop, and Holliday junction) and both linear single-stranded and double-stranded DNA, produced by annealing partially complementary oligonucleotides. All contained a common 5′-^32^P end-labeled DNA strand (Fig 5C and oligonucleotide #1 S4 Table). We found that SHOC1^570-1111^ binds to single-stranded DNA, Holliday junction and D-loop substrates most efficiently, followed by the splayed arm and linear duplex, as indicated by the appearance of slow-migrating products during neutral PAGE (Fig 5C and 5D).

The (HhH)_2_ motif participates in DNA binding of other DNA repair enzymes. For example, XPF/MUS81 endonucleases recognize the junction of single-stranded/double-stranded in a resected double-strand DNA molecule with a precise polarity [24], and the major determinant of binding branched DNA junctions localizes to the (HhH)_2_ domain [43, 44]. This globular domain contains two copies of a sequence-independent HhH DNA-binding motif, which are bridged by a fifth connecting helix [45, 46] (Fig 5E, top panel). (HhH)_2_ domains use their hairpins to bridge across the minor groove face of duplex DNA by engaging a backbone phosphate from each strand separated by three base pairs [44, 47]. We tested the effect of deleting the (HhH)_2_ motifs (purified SHOC1^570-1041^ mutant in a SDS-PAGE gel is shown in Fig 5E) on SHOC1 DNA binding. Deletion of both HhH domains substantially reduced the ability of SHOC1 to bind single-stranded DNA (Fig 5E). We do not think this is caused by alterations in SHOC1 conformation because we observed similar profiles when thermal scanning with an environment-sensitive dye probe was performed on SHOC1^570-1111^ and SHOC1^570-1041^, suggesting no gross changes in protein stability (S7C Fig). In sum, we observed that recombinant mouse SHOC1 has a strong preference for branched structures and single-stranded DNA and the SHOC1 (HhH)_2_ domain plays a role in efficient DNA binding.

### Recombinant SHOC1 lacks *in vitro* endonuclease activity

The canonical GDX_n_ERKX_3_D nuclease active site motif found in human XPF and MUS81 is not conserved in human SHOC1 (ERKX_3_E), similar to human FANCM protein (ERRX_3_E). As the potential nuclease activity of SHOC1 should allow processing of intermediates of the recombination pathway we tested for the ability of recombinant SHOC1^570-1111^ to endonucleolytically cleave Holliday junction DNA structures (see Methods) used for the DNA binding assay (Fig 5) by evaluating the appearance of fast-migrating products in neutral PAGE [48] after or before incubation with proteinase K and SDS (S8A Fig). We also analyzed the ability of SHOC1^570-1111^ to process double-stranded supercoiled DNA substrates (ΦX174 RFI) (S8B Fig). Thus far, we have not detected nuclease activity *in vitro* using human recombinant purified SHOC1^570-1111^ (S8 Fig). This positions SHOC1 (and presumably FANCM) as unique members in the XPF/MUS81 family that exhibit no nuclease activity [49, 50]. Although SHOC1^570-1111^ exhibits DNA binding activity, we acknowledge that the lack of *in vitro* nuclease activity may be an artifact of the recombinant protein we used in these assays.

**Fig 6:**
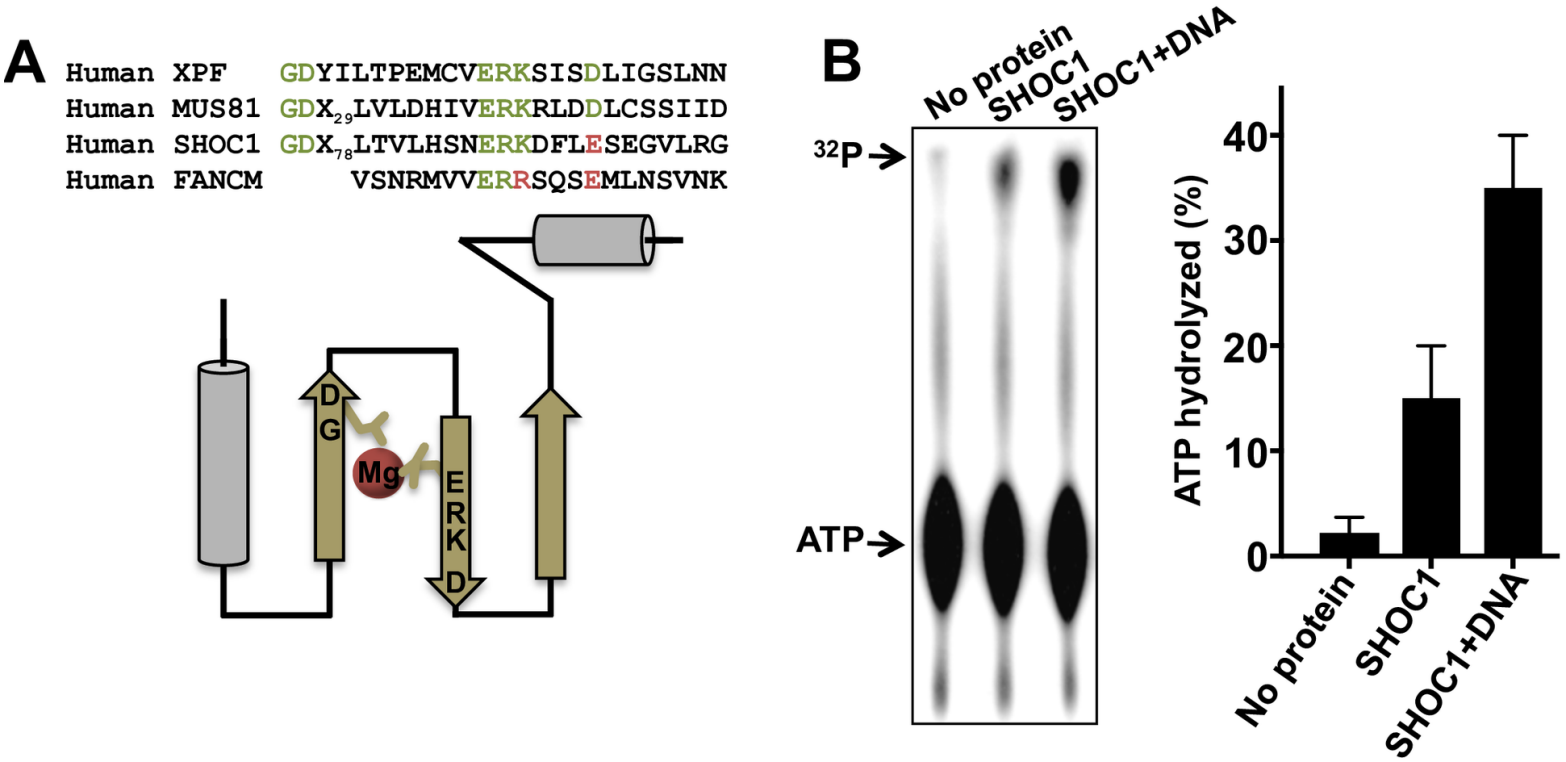
ATPase activity of purified human SHOC1. (A) The human SHOC1 endonuclease-like domain diverges from a canonical XPF-like sequence. (B) Purified human SHOC1^570-1111^ exhibits an ATPase activity, which is stimulated by DNA. Left panel, separation of the products of ATP hydrolysis by thin layer chromatography. Right panel, quantitation of the results (n = 3, mean ± SD).

Although SHOC1 ERKX_3_E may be nucleolytically inactive, the acidic residues within this motif may still coordinate a divalent metal ion (Fig 6A), which may be required for structural stability and/or participating in other roles proposed for these domains, such as binding single-stranded DNA or targeting the protein to defined DNA structures [43].

We also observed that SHOC1 has an associated ATPase activity, which is stimulated in the presence of single-stranded plasmid DNA (Fig 6B). We note that FANCM also binds single-stranded DNA and exhibits ATPase activity [51], which may be related to the ability of FANCM to translocate along DNA [50]. Future studies will determine if SHOC1 shares this type of interaction with DNA.

### SHOC1 interacts with TEX11, a homologous recombination-promoting factor

**Fig 7:**
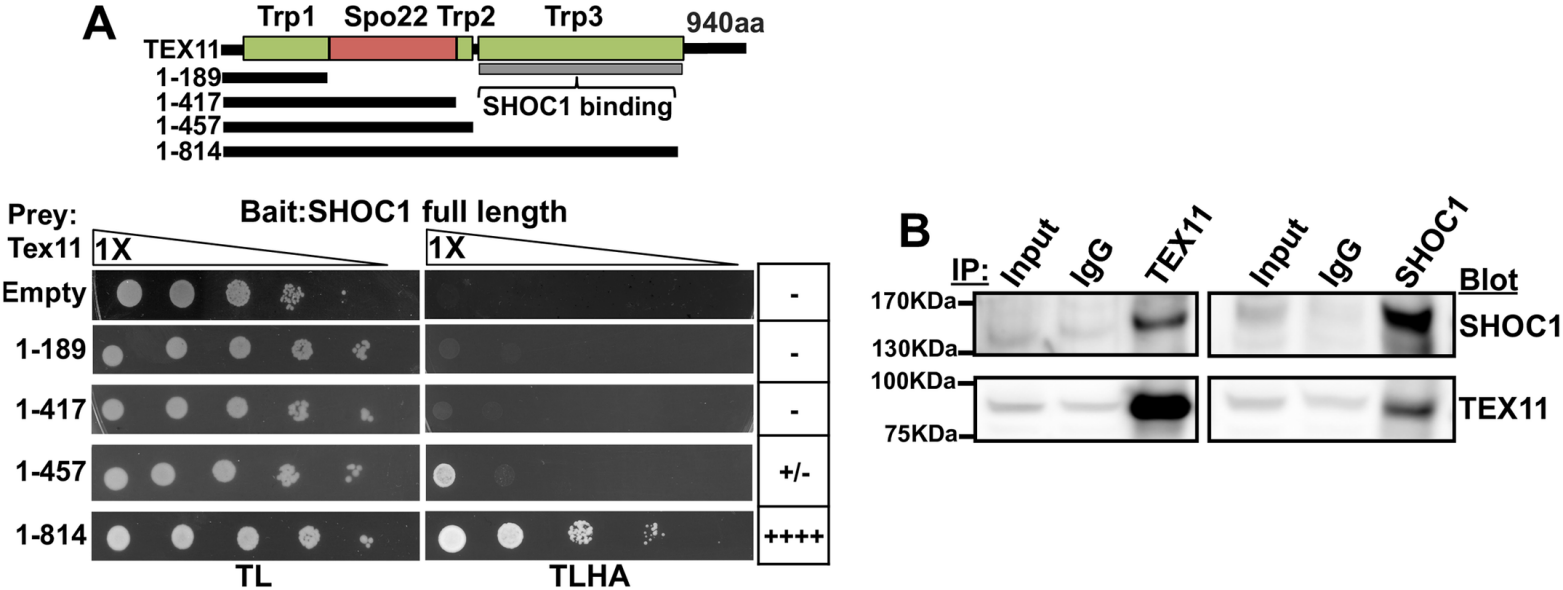
SHOC1 interacts with TEX11. (A) Direct yeast two-hybrid assay showing the interaction between the C-terminal portion of human TEX11 and human SHOC1. Predicted domains within TEX11 are also shown. (B) Co-immunoprecipitation of TEX11 and SHOC1 from total testis extract of 13 day-old mice. Samples in IgG, SHOC1, and TEX11 lines are 6X compared to input.

Although our work strongly implicates SHOC1 in the recombination process, how SHOC1 integrates with described recombination factors is poorly understood. Prompted by similarities between the meiotic phenotype of mice deficient in SHOC1 or TEX11 [30, 31] and prominent SHOC1-TEX11 co-localization during early pachytene spermatocytes (Fig 1D), we analyzed their interaction by a direct yeast-two hybrid assay. Our results indicate that a C-terminal portion of TEX11 corresponding to a TRP-like domain, a ubiquitous protein interaction domain that adopts a modular antiparallel array of α-helices [52], interacts with SHOC1 (Fig 7A). We obtained further evidence for a SHOC1-TEX11 interaction by co-immunoprecipitation experiments using testis extract and specific polyclonal antibodies against SHOC1 and TEX11 (Fig 7B and S9 Fig). We conclude that human SHOC1 forms complexes with TEX11, a protein known to promote recombination-dependent DSB repair and CO formation [30, 31, 53].

## Discussion

We show that purified recombinant SHOC1 can bind DNA *in vitro* with a strong preference for DNA branched structures. In agreement, immunolocalization experiments reveal that SHOC1 concentrates to a subset of recombination sites that are shown to be a key intermediates in the mid-stage of the CO formation process. Further, SHOC1 deficient spermatocytes are deficient in CO formation. Although most *Shoc1*^*hyp/hyp*^ spermatocytes exhibit a comparable number of initial recombination events to wild-type mice, they show a reduced number of MLH1 foci, reduced chiasma formation, and arrest at metaphase I with lagging chromosomes and subsequent apoptosis. In sum, the evidence suggests that also in mammals SHOC1 acts through a major pathway for CO formation. However, this CO deficient phenotype seems to be less severe than that observed in yeast and Arabidopsis. *Zip2* mutants showed 40–50% reduction in the number of COs with respect to wild type; and defects in CO formation were even higher at specific hotspots [20–22]. In Arabidopsis, Shoc1 mutant chiasmata frequency per cell (0.8–1.27) are significantly lower (80–90%) compared to wild type (7.8–9.2) [29]. The observed disparity between mouse and the yeast and plant counterparts may be explained by interspecies differences in the role of SHOC1. However, it is also possible that the moderate phenotype observed in mice is caused by the incomplete ablation of SHOC1.

Chromosome associations are also affected in mouse *Shoc1* mutant spermatocytes. Pairing and initial synapsis is apparently normal but synapsis is incomplete for most chromosomes in *Shoc1*^*hyp/hyp*^ spermatocytes. This effect of *Shoc1* mutation is similar to that described in the yeast *Zip2* mutant but not in Arabidopsis. In yeast, chromosomes are homologously paired but not synapsed [19] and synaptonemal complex formation is abnormal in a *Zip2* mutant [22]. In Arabidopsis, Shoc1 mutant meiocytes at pachytene stages resemble those from wild type mice, with apparent normal homologous chromosome pairing and synapsis.

Our findings in mice has several points in common with a recent work in yeast [54] showing that Zip2 and Spo16 form an Xpf-Ercc1-like complex that drives intermediate recombination products toward crossover. Remarkably, both Shoc1 and Zip2-Spo16 recognizes DNA branches structures, are important for formation of normal numbers of meiotic crossovers, and interact with Tex11/Zip4, another protein of the ZMM group important for crossover formation.

### What is the role of SHOC1?

**Fig 8:**
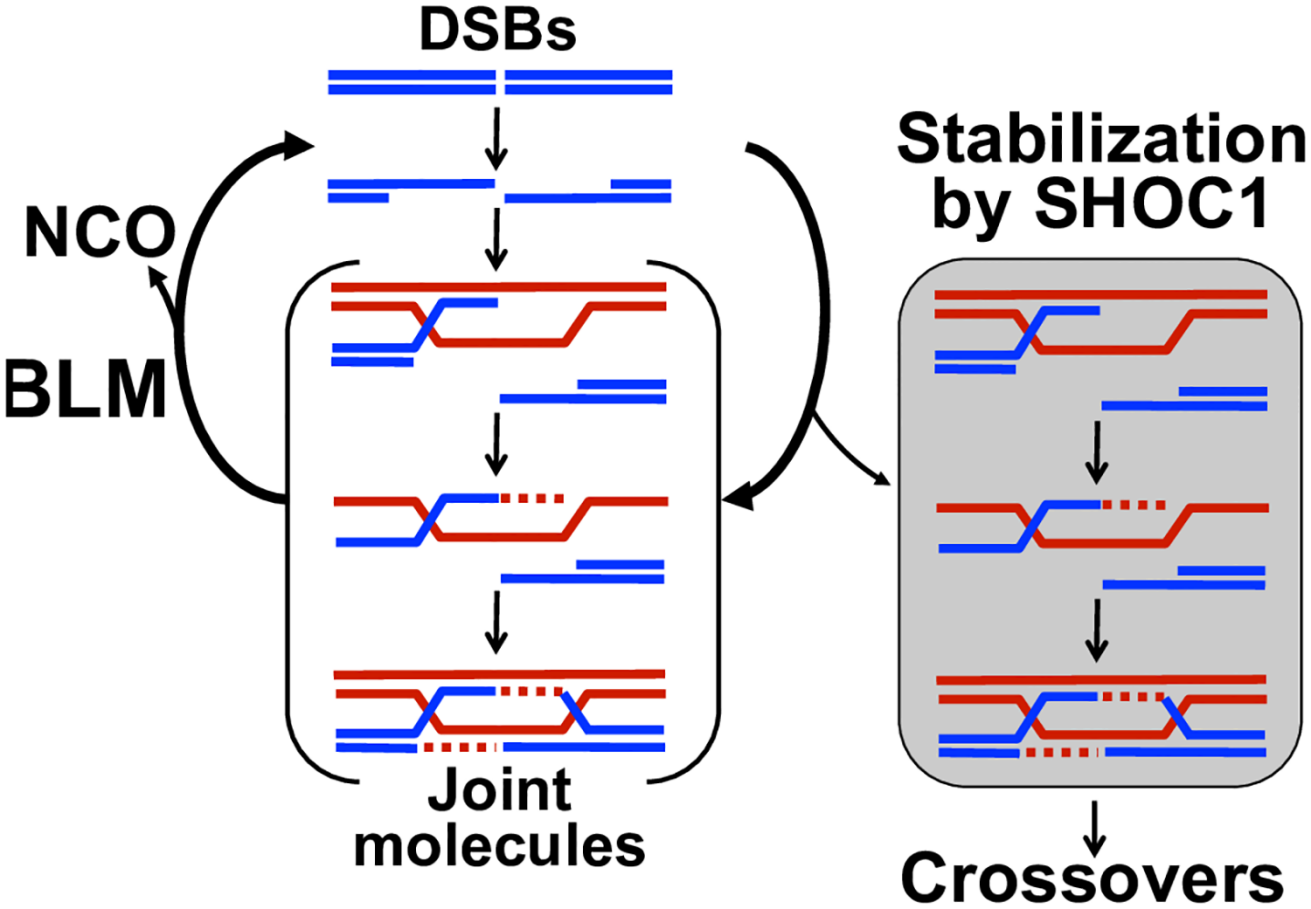
Proposed function of SHOC1 in meiotic recombination. SHOC1 selectively binds and protects branched recombination intermediate structures from dissociation. BLM: Bloom Syndrome RecQ like Helicase.

How could SHOC1 promote CO formation? Using protein sequence and structural analyses, we and others found that human and mouse SHOC1 have putative ERCC4-like nuclease motifs with a GEX_22_ERKX_3_E (DRK for mouse) sequence significantly similar to members of the XPFG/MUS81 family of nucleases [23]. Two well-defined HhH domain segments follow this motif. Although conservation of the ERCC4-(HhH)_2_ core suggests potential nuclease activity for SHOC1, the ERCC4-like domains in mouse and human SHOC1 diverge from the canonical sequence. Since in our hands recombinant human SHOC1^570-1111^ apparently lacks *in vitro* nuclease activity, our results argue against a possible role for Shoc1 in cleaving recombination intermediates to produce COs. This conclusion is further supported by divergent catalytic sites also found in Arabidopsis and yeast [23]. Instead, we argue that SHOC1 binds DNA branched structures to stabilize them. We propose that SHOC1 binding protects against dissociation by anti-crossover activities, such as BLM (Bloom syndrome protein) functions that promote disassembly of D-loops catalyzed by RAD51 [20, 55, 56] (Fig 8).

Intriguingly, in our *in vitro* DNA binding assay, SHOC1 binds single-stranded DNA with high affinity and exhibits ATPase activity. These biochemical characteristics have also been found in human recombinant FANCM-FAAP24, in which these are important features to target the protein to DNA structures containing single-stranded DNA [25].

Most ERCC4-(HhH)_2_ members of the XPF/MUS81 protein family exist in their functional state as heterodimers in eukaryotes or as homodimers in archaea. In agreement, Arabidopsis Shoc1 interacts with Ptd1, a protein with sequence similarity to Ercc1 and that contributes to meiotic CO formation [29]. Our current efforts to identify a SHOC1 interaction partner (screening of a testis specific yeast-two-hybrid library with SHOC1 as a bait, and SHOC1 immunoprecipitation followed by mass spectroscopy) with structural characteristics or an equivalent role as Ptd1 have been fruitless. In this case, we speculate that SHOC1 in mammals could function alone. Indeed, in our assays SHOC1^570-1111^ acts in its monomeric form (S7A and S7B Fig) and seems proficient in binding branched structures. Alternatively, SHOC1 may interact with known ERCC1-like proteins (i.e. EME1, FAAP24) or with an unidentified protein, but their interaction may be transient making detection difficult. Future work will investigate these possibilities.

### The connection of SHOC1 with chromosome structure

We observed that SHOC1 interacts with TEX11, the mammalian homolog of budding yeast Zip4, a large TRP-repeat protein required for normal synapsis and CO formation in mice [21, 30, 31]. TEX11 localizes as numerous foci along synaptonemal complexes during zygotene and pachytene stages and colocalizes with intermediate stage recombination components RPA and MSH4 [31]. TEX11 interacts with SYCP2, a component of the synaptonemal complex [31, 57], which suggests that TEX11 may provide a physical link between SHOC1, chromosome synapsis, and CO formation. TEX11 may also act as a scaffold protein to assist recruitment and/or stabilization of SHOC1 and other ZMM proteins to recombination sites.

## Materials and methods

### Ethics statement

Experiments conformed to relevant regulatory standards guidelines and were approved by the Oklahoma Medical Research Foundation-IACUC (Institutional Animal Care and Use Committee). Protocol number 17–25.

### Generation of SHOC1-deficient mice

To generate SHOC1 deficient mice we designed a gene trap vector that replaced coding exons 2–20 of *Shoc1*: 1) The BAC RP23-94F24 was used to amplify the DNA region used as an insertion site for the functional cassette and the stretches to be subcloned in order to confirm correctness of the initial BAC. 2) The PGK-gb2-neomycin marker cassette was inserted into the identified BAC clone by Red/ET recombination. Accurate performance of Red/ET recombination was verified by PCR. 3) A BAC fragment of approximately 14kb was subcloned into a minimal vector pMV (colE1 origin of replication; ampicillin resistance marker) by Red/ET recombination. 4) Functional regions were verified by sequencing. Knockout mouse production was performed by the Texas A&M Institute for Genomic Medicine according to the following scheme: (i) vector DNA purification and linearization, (iii) electroporation of ES cells followed by cell isolation and selection, (iv) generation of chimeras by ES cell injection into C57BL/6 blastocysts, (v) breeding chimeras with wild-type C57BL6N females to obtain germline F1 heterozygotes that were genotyped by PCR (see below). Further crosses of heterozygous mice generated *Shoc1*^*+/+*^ and *Shoc1*^*+/-*^ mice. *Shoc1*^*-/-*^ mice were not detected suggesting embryonic lethality.

The procedure for the generation of SHOC1 deficient mice, in which the gene-trapping cassette (LacZ-Neo) was designed to replace exons 2–4 and prevent translation of downstream fusion transcripts, is detailed in S3F Fig. *Shoc1*^*hyp/wt*^ ES cells were injected into C57BL/6 blastocysts to create chimeric mice, which were bred with C57BL/6 mice to generate *Shoc1*^*hyp/wt*^ heterozygous mice. Further crosses of heterozygous mice resulted in the generation of *Shoc1*^*hyp/hyp*^ mice, which were used for phenotypic analyses.

### Genotyping of mice by PCR

The genotyping of *Shoc1* mice carrying a deletion in exons 1–20 was carried out by PCR (KAPA2G Fast multiplex PCR kit) using oligonucleotides 5′F (S4 Table) and R2 to amplify the wild type allele (240 bp), and 5′F and SAPR3 to amplify the mutant allele (425 bp). The cycling conditions were: 95°C 3 min; 95°C 15 sec, 57°C 30sec, 72°C 60sec for 35 cycles and 72°C 3 min.

The genotyping for *Shoc1*^*hyp/hyp*^ mice was carried out by PCR (KAPA2G Fast multiplex PCR kit) using oligonucleotides F2 (S4 Table) and Neo to amplify the wild type allele (616 bp), and Neo and R1 to amplify the mutant allele (705 bp). The cycling conditions were: 95°C 30 sec; 95°C 10 sec, 65°C 30 sec, 72°C 60 sec for 35 cycles; 72°C 5 min.

### Real-time RT-PCR

Total RNA was isolated from adult testis with the RNeasy Mini Kit (Qiagen). RNA (2.0μg) was oligo-dT primed and reverse-transcribed with the High capacity RNA-to-cDNA kit (Applied Biosystems). Three different exon boundaries of *Shoc1* were amplified using Power Sybr Green PCR Master Mix (Applied Biosystems) with specific primers (S4 Table). The cycling conditions were: 95°C 10 min, 95°C 10 sec, 60°C 30 sec, for 45 cycles. A melt curve was performed at 95°C for 10 sec and 65°C to 95°C in increments of 0.5°C for 5 sec. Experiments were performed in a Light Cycler 480 II (Roche) and results analyzed with the software Light Cycler 480 SW 1.5.1. Gene expression was normalized with respect to wild type with wild type expression levels considered to be 1.

### Histological analysis

For histological examination, testes and ovaries were removed and fixed overnight in 10% neutral-buffered formalin (Sigma). Serial sections from paraffin-embedded testes or ovaries were positioned on microscope slides and analyzed using either hematoxylin & eosin staining or a TUNEL assay (Roche).

### Cytology

We used established experimental approaches to visualize chromosomes in chromosome surface spreads [58]. Incubation with primary antibodies occurred for 12h at 4°C in 1× PBS plus 2% BSA. To detect SYCP1 and SYCP3, we used polyclonal rabbit anti-mouse SYCP1 (1:200, Novus Biologicals) and polyclonal mouse anti-mouse SYCP3 (1:300, Abcam). Other primary antibodies used in this study include: monoclonal mouse anti-mouse γH2AX at (1:500, Millipore), polyclonal rabbit anti-mouse RAD51 (1:200, Santa Cruz Biotechnology), polyclonal goat anti-human DMC1 at (1:300, Santa Cruz Biotechnology), polyclonal rabbit anti-mouse MSH4 (1:200, AbCam), polyclonal rabbit anti-mouse TEX11 (1:100) [31], and monoclonal mouse anti-mouse MLH1 (1:50, BD Pharmingen). Polyclonal SHOC1 antibodies were generated in rabbits (Cocalico Biological Inc.) immunized with a bacterial expressed, urea denatured HA-purified mouse SHOC1 fragment isolated from inclusion bodies (540–1250 aa, cDNA cloned in pET15b) and by affinity chromatography on a SHOC1 protein fragment (amino acids 540–1250). Slides were incubated with primary antibodies followed by three washes in 1X PBS and then incubated for 1 h at room temperature with secondary antibodies. A combination of fluorescein isothiocyanate (FITC)-conjugated goat anti-rabbit IgG (Jackson laboratories) with rhodamine-conjugated goat anti-mouse IgG and Cy5-conjugated goat anti-human IgG each diluted 1:250 were used for simultaneous triple immunolabeling. Slides were subsequently counterstained for 3 min with 2 μg/ml DAPI containing Vectashield mounting solution (Vector Laboratories) and sealed with nail varnish. Foci were counted manually and only those that co-localized with the chromosome axes were considered. We used Axiovision SE 64 (Carl Zeiss) for imaging acquisition, visualization for foci counting, and processing.

### Proteins and DNA

For DNA binding, all oligonucleotides (IDT, Inc., see S1 Table) were purified, ^32^P labeled, annealed and stored as described previously [59]. DNA branched substrates were formed by the annealing of equimolar amounts of two or more oligonucleotides followed by purification in native polyacrylamide gels. The oligonucleotides used are: single-stranded (#1), linear duplex (#1, #5), splayed arm (#1, #4), Holliday junction (#1, #2, #3, #4), and D-loops (#1, #6, #7) (S4 Table).

### DNA binding assay

SHOC1^570-1111^ was incubated with 10 μM of ^32^P-labeled DNA in the following buffer: 20 mM Tris-HCl (pH 7.4), 1 mM DTT, 2 mM MgCl_2_ and 100 mM NaCl in a volume of 20 μl for 10 min at 37°C. The samples were mixed with 3 μl of loading buffer (30% sucrose, 0.1% bromphenol blue) and analyzed by electrophoresis in 8% polyacrylamide gels in 1× TAE buffer at 5 V/cm for 5 h. The formation of nucleoprotein complexes and mobility shift of labeled DNA were imaged using autoradiography film or a BAS 2500 Bio-imaging Analysis System (Fuji Medical System).

### Nuclease assay

Reactions were performed in 10 μl containing 10 μM ^32^P-labeled synthetic nucleotide substrate DNA (Holliday junction) in phosphate buffer: 60 mM sodium phosphate (pH 7.4), 1 mM DTT, 0.1 mg/mL BSA, and 5 mM Mg(OAc)_2_. After incubation of SHOC1^570-1111^ (1μM) at 37°C for periods of time up to 30 min, DNA products were deproteinized (not in controls) for 15 min at 37°C using 2 mg/ml proteinase K and 0.4% SDS. Products were analyzed by 10% neutral PAGE followed by autoradiography. Cleavage of ΦX174 DNA (10 μM, Biolabs) was carried out in the above phosphate buffer. Reactions were pre warmed to 37°C and initiated by enzyme addition, and, after different periods of time (up to 30 min), reactions were deproteinized. DNA products were resolved by 1% agarose gel electrophoresis, stained with ethidium bromide and imaged.

### ATPase assay

Reactions were assembled by mixing 250 nM SHOC1^570-1111^ in a 10 μl solution containing 50 mM Tris·HCl, pH 7.5, 5 mM MgCl_2_, 0.1 mg/ml BSA, 1 mM ATP, 16 nM [γ-^32^P]ATP and 1 mM DTT. To test the impact of DNA, we added 0.75 μM ΦX174 virion single-stranded DNA (New England Biolabs) to the reaction. In control experiments SHOC1^570-1111^ was omitted. Reactions were allowed to proceed for 10 min at 37°C and were stopped by the addition of 10 mM EDTA. One μl of each reaction mixture was spotted on Silica gel 60 F254 TLC aluminum sheets and developed in a solution containing dioxan, isopropanol, 28% ammonia and water (v/v, 4:2:3:4). TLC plates were imaged using autoradiography film and scanned for quantification.

### Yeast two-hybrid analysis

Human TEX11 truncations (DNA encoding amino acids 1–189, 1–417, 1–457, and 1–814) were cloned into pGADT7-AD (Prey, Clontech), to produce fusions to the Gal4 DNA-binding and activation domains. A plasmid containing full-length human SHOC1 was constructed by cloning the appropriate PCR product into pGBKT7 (Bait, Clontech). All fusions were confirmed by sequencing. Two-hybrid assays were conducted in the AH109 strain background. After mating, colonies containing both plasmids were selected using media lacking tryptophan and leucine. Interactions between partners were assayed by growth on synthetic media lacking tryptophan, leucine, adenine and histidine. Transformations were carried out according to the matchmaker kit manual (BD Biosciences).

### Western blot and co-immunoprecipitation

Testes were dissected in 1X PBS and cells lysed using IP buffer (0.5% Triton X-100, 50 mM Tris-HCl (pH 7.4), 150 mM NaCl, 3 mM MgCl_2_, 10% Glycerol, 1 mM DTT, EDTA-free protease inhibitors, and benzonase). For Western blots 1 volume of sample buffer was added (4% SDS, 160 mM Tris-HCl, (pH 6.8), 20% glycerol, 100 mM DTT, and 0.005% bromphenol blue). For immunoprecipitation, samples were treated with benzonase (15 min on ice) and after centrifugation (10,000 RPM for 1min), proteins from the soluble fraction were immunoprecipitated with antibodies (10 μg) pre-bound with protein A ultralink resin beads (Thermo Fisher Scientific). After rotation at 4°C for 6h, the beads were washed four times with ice-cold IP buffer, and bound proteins were eluted by boiling for 5 min with SDS-PAGE sample buffer. Proteins were separated by 4–15% gradient SDS-PAGE under reducing conditions and transferred to nitrocellulose membranes. The blots were probed with individual primary antibodies as indicated, and then incubated with HRP-conjugated donkey anti-mouse or rabbit secondary antibodies as required. In all blots, proteins were visualized by chemiluminescence in a C-600 Imager (Azure Biosystems).

### Protein thermal shift

Temperature dependent protein denaturation in presence of a hydrophobic sensitive dye was performed as directed by the manufacturer instruction guide (Protein Thermal Shift Dye Kit, part number 4461146, Roche). We used 3μg of each SHOC1^570-1111^ and SHOC1^570-1041^ in a total volume of 20 μl.

### Statistical reporting

Statistical analysis methods are described in the text or Figure legends. GraphPad Prism (version 6.0f) package was used for generation of graphs and all statistical analyses.

## Supporting information

S1 Fig SHOC1 immunostaining of spermatocyte chromosome spreads from wild type and *Shoc1*^*hyp/hyp*^ mice. Wild type and *Shoc1*^*hyp/hyp*^ chromosome spreads immunostained with SHOC1 antibodies. Note the lack of immunostaining in *Shoc1*^*hyp/hyp*^ spermatocytes.
(TIF)

S2 Fig Co-localization of SHOC1 immunosignal with cytological markers of recombination in wild type spermatocytes. Wild type mouse spermatocytes at different stages of prophase I immunostained with anti-SYCP3, anti-SHOC1, anti-TEX11, anti-MSH4, anti-DMC1, and anti-MLH1 antibodies are shown. Chicken SYCP3 antibodies were used to mark the chromosome cores.
(TIF)

S3 Fig Generation of SHOC1 deficient mice and analysis of embryonic lethality. (A) *Shoc1* gene targeting design for mutants in which 2–21 exons were deleted. (B, C) Genotype analysis of adult mice (B) and 9.5dpc embryos (C) from Shoc1 heterozygous crosses. Note absence of mice carrying knockout genotype. (D) Expression pattern of *Shoc1* in selected adult tissues. Tissue distribution expression data were obtained from the Mouse Genomic Informatics (MGI) mouse genome database (MGD) mouse genome database. Ensembl IDs are used for genes. (E) Expression pattern of *Shoc1* in selected tissues during embryonic development. Data were obtained from the RNA profiling data sets generated by the mouse ENCODE transcriptome project (BioProject PRJNA66167). CNS, central nervous system. (F) *Shoc1* gene targeting design for mutants in which 2–4 exons were deleted. Oligonucleotides and PCR products used for confirmation of vector construction using long distance PCR also shown.
(PDF)

S4 Fig Confirmation of the Neo-PGK-gb2 cassette insertion at the *Shoc1* locus. (A) *Shoc1* gene targeting design for mutants in which 2–21 exons were deleted. (B) Results obtained using long PCR-based analysis of the Neo-PGK-gb2 insertion in *Shoc1* heterozygous mice. The primers used are indicated in A. (C) Sequence of two PCR fragments (F2-R2, and F4-R4 primers) shown in B. Sequenced portions are underlined
(PDF)

S5 Fig Histological sections of wild type and *Shoc1*^*hyp/hyp*^ ovaries. Hematoxylin and eosin stained ovary paraffin sections from wild type and mutant mice shown. Follicles, F. Corpora lutea, Cl. Quantitation of number of follicles and corpora lutea per analyzed H&E ovary section. One middle section of each analyzed ovary (obtained from at least three different 35 day old mice) was used for quantitation.
(PDF)

S6 Fig Characterization of the SHOC1 sequence. (A) SHOC1 sequence alignment between related mammalian species at the putative Shoc1 XPF-like domain. (B) Prediction of structural disordered areas of human SHOC1 using the GlobePlot2 and PrDOS prediction algorithms.
(PDF)

S7 Fig Structural characterization of recombinant human SHOC1^570-1111^. Size determination of SHOC1^570-1111^ by chemical cross-linking (A) and size exclusion chromatography (B). 8μM SHOC1 was incubated with the indicated amounts of suberic acid bis(N-hydroxysuccinimide ester) (DSS) for 10 min at room temperature. The samples were resolved in 4–12% gradient SDS-PAGE gels, and the proteins were stained with Coomassie Brilliant Blue. Determination of the Stoke’s radius for SHOC1 (B) was calculated by gel filtration chromatography on Superdex 200. The column was calibrated using different molecular weight markers as indicated in Buffer (Tris-HCl 7.4, NaCl 250mM, and glycerol 10%) and the column outlet was monitored at 280nm. The estimated molecular weight of SHOC1 (125.84 kDa) was calculated by the formula Y = -746*X+1215 obtained from a regression plot. (C) Thermal denaturation curve in presence of a thermal sensitive dye for SHOC1^570-1111^ and SHOC1^570-1041^.
(PDF)

S8 Fig Purified recombinant SHOC1^570-1111^ apparently lacks nuclease activity. Human recombinant SHOC1^570-1111^ was incubated with oligonucleotide-based Holliday junction structures and developed in TAE-polyacrylamide gels (A) or supercoiled dsDNA φX174 (RFI) and developed in 1% agarose gels (B) as indicated in Materials and Methods.
(PDF)

S9 Fig SHOC1 interacts with TEX11. The product of co-immunoprecipitation of TEX11 and SHOC1 from total testis extract of 13 day-old mice is shown. SHOC1, and TEX11 lines are 8X compared to input. Signal acquisition time in the upper and lower panel is not equal.
(PDF)

S1 Table Measurement of seminiferous tubule diameter in wild type and *Shoc1*^*hyp/hyp*^ mice. (PDF)

S2 Table Product of crosses between females wild type and Shoc1^hyp/hyp^ with wild type males. (PDF)

S3 Table Measurement of homologous chromosome length in Shoc1^hyp/hyp^ spermatocytes. (PDF)

S4 Table Sequences of oligonucleotides used in this study. (PDF)
