# Supplementary figures and images for: SHOC1 is a ERCC4-(HhH)2-like protein, integral to the formation of crossover recombination intermediates during mammalian meiosis
Source: PLoS Genet. 2018 May 9;14(5):e1007381. doi: 10.1371/journal.pgen.1007381 (PMC5962103; doi:10.1371/journal.pgen.1007381)

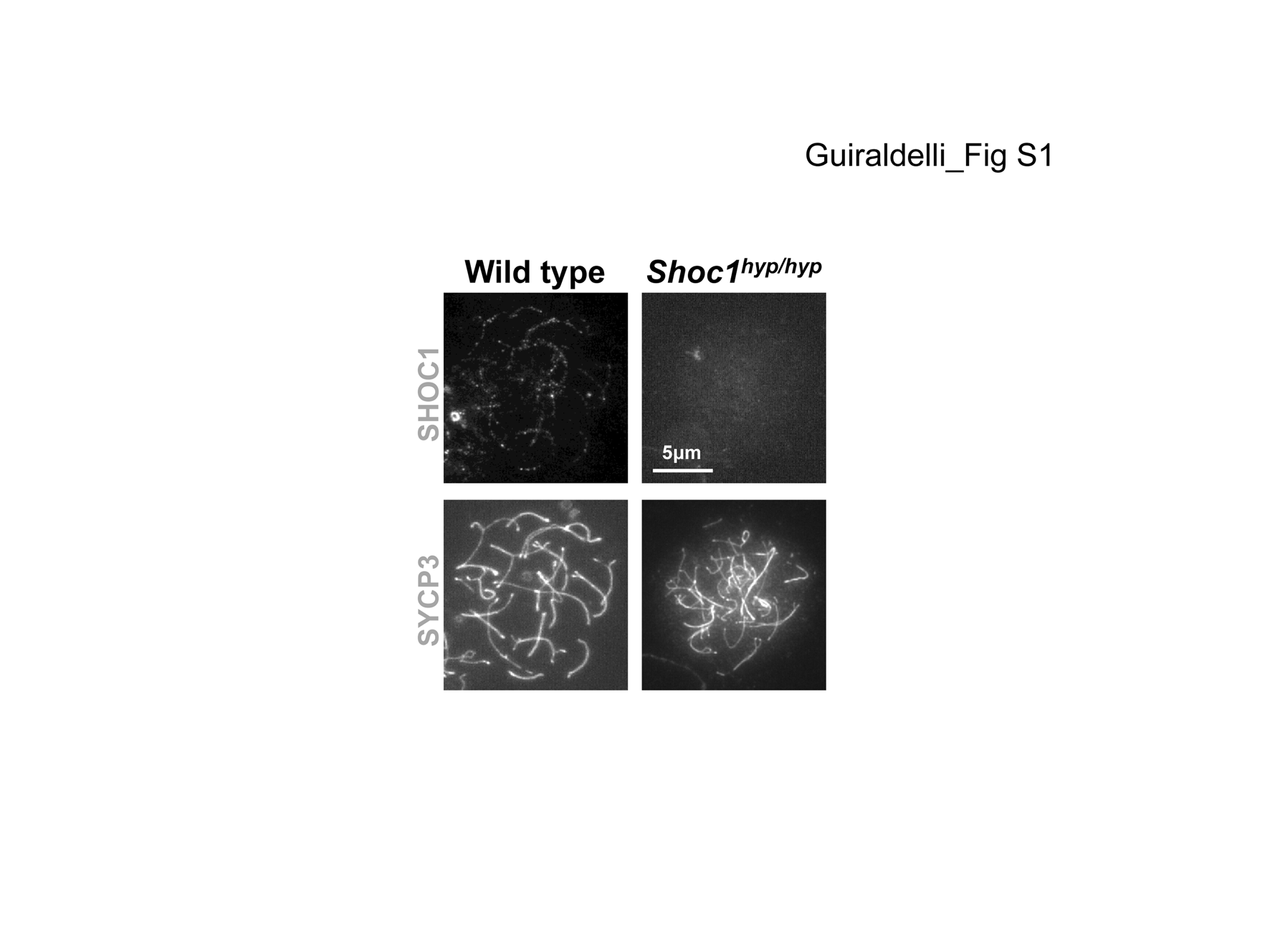

Supplement: S1 Fig — Wild type and Shoc1hyp/hyp chromosome spreads immunostained with SHOC1 antibodies. Note the lack of immunostaining in Shoc1hyp/hyp spermatocytes. (TIF) [file pgen.1007381.s001.tif]

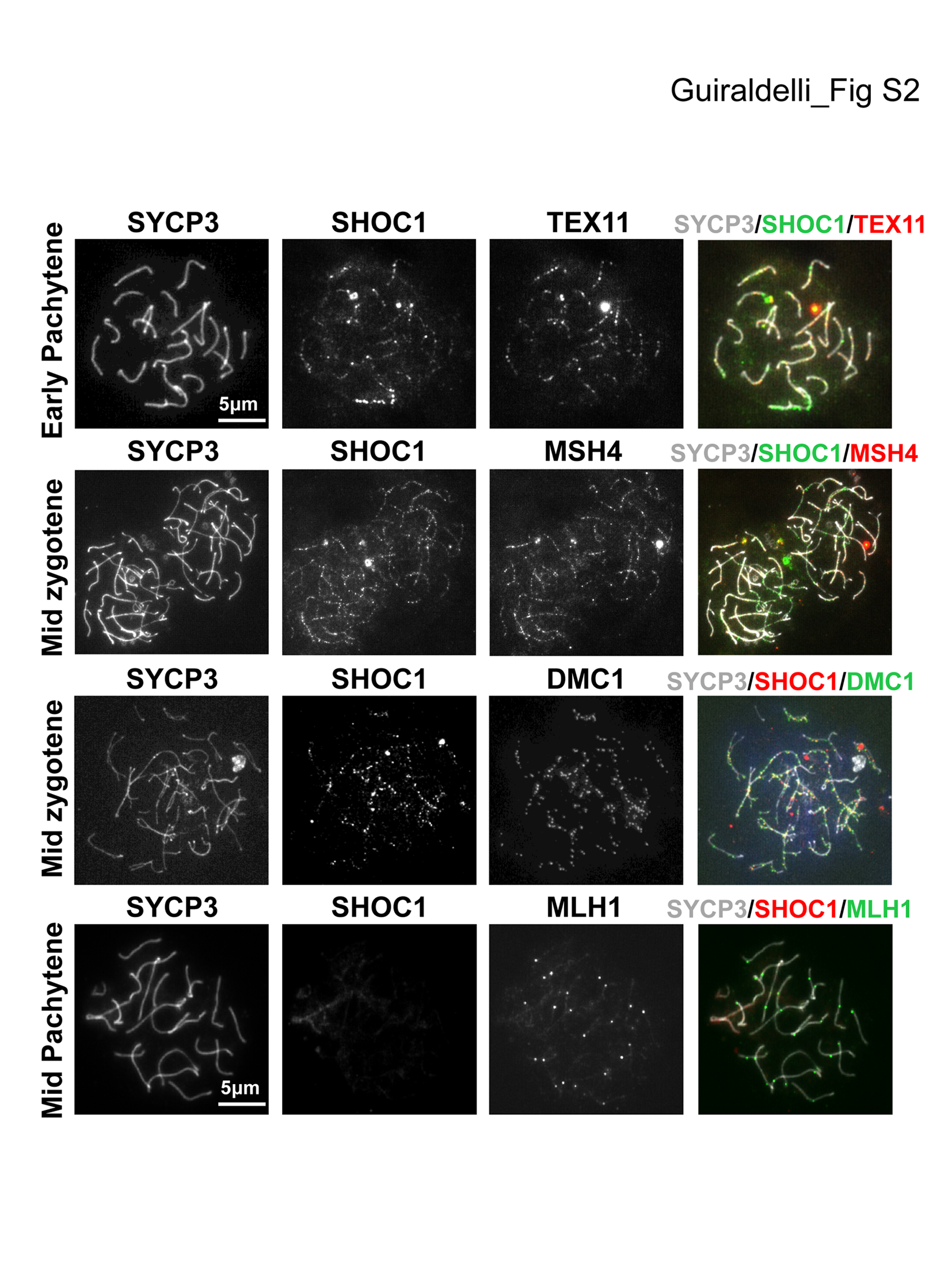

Supplement: S2 Fig — Wild type mouse spermatocytes at different stages of prophase I immunostained with anti-SYCP3, anti-SHOC1, anti-TEX11, anti-MSH4, anti-DMC1, and anti-MLH1 antibodies are shown. Chicken SYCP3 antibodies were used to mark the chromosome cores. (TIF) [file pgen.1007381.s002.tif]

Wild type

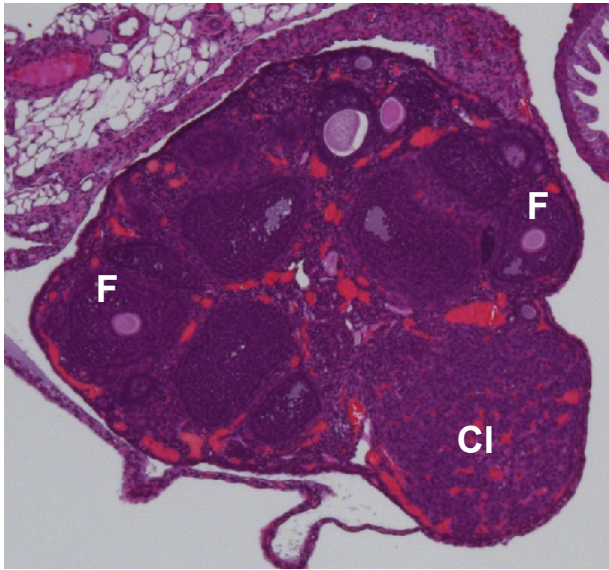*Shoc1*<sup>hyp/hyp</sup>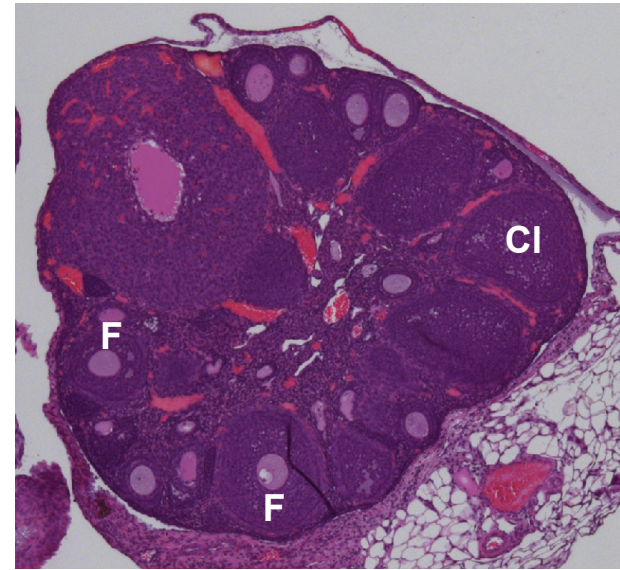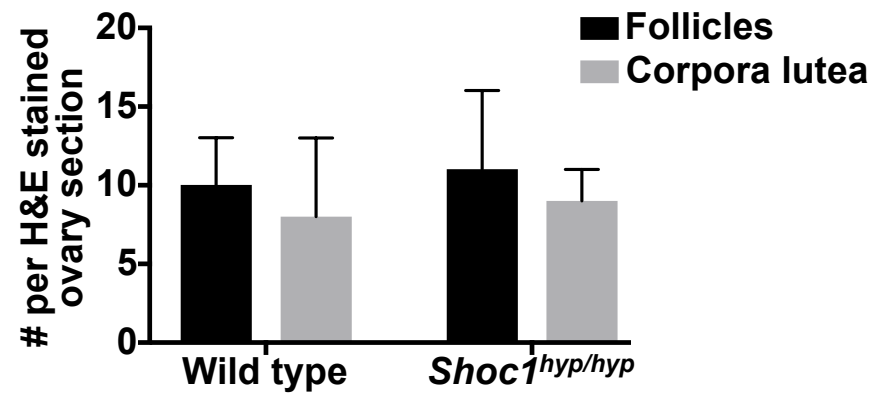

Supplement: S5 Fig — Hematoxylin and eosin stained ovary paraffin sections from wild type and mutant mice shown. Follicles, F. Corpora lutea, Cl. Quantitation of number of follicles and corpora lutea per analyzed H&E ovary section. One middle section of each analyzed ovary (obtained from at least three different 35 day old mice) was used for quantitation. (PDF) [file pgen.1007381.s005.pdf]

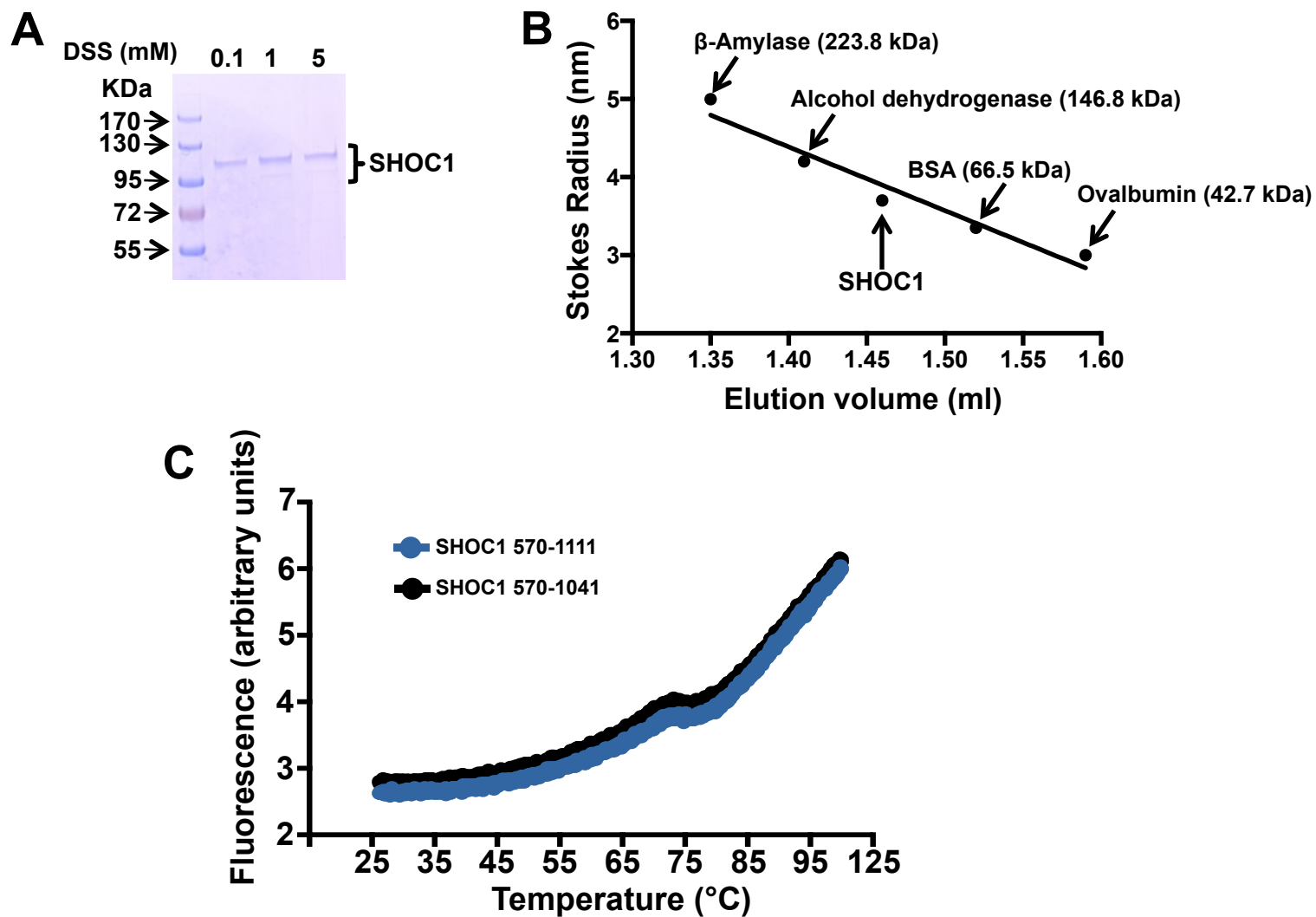

Supplement: S7 Fig — Size determination of SHOC1570-1111 by chemical cross-linking (A) and size exclusion chromatography (B). 8μM SHOC1 was incubated with the indicated amounts of suberic acid bis(N-hydroxysuccinimide ester) (DSS) for 10 min at room temperature. The samples were resolved in 4–12% gradient SDS-PAGE gels, and the proteins were stained with Coomassie Brilliant Blue. Determination of the Stoke’s radius for SHOC1 (B) was calculated by gel filtration chromatography on Superdex 200. The column was calibrated using different molecular weight markers as indicated in Buffer (Tris-HCl 7.4, NaCl 250mM, and glycerol 10%) and the column outlet was monitored at 280nm. The estimated molecular weight of SHOC1 (125.84 kDa) was calculated by the formula Y = -746*X+1215 obtained from a regression plot. (C) Thermal denaturation curve in presence of a thermal sensitive dye for SHOC1570-1111 and SHOC1570-1041. (PDF) [file pgen.1007381.s007.pdf]

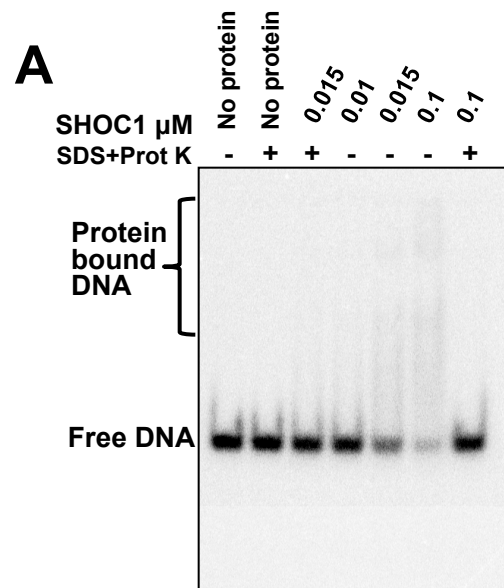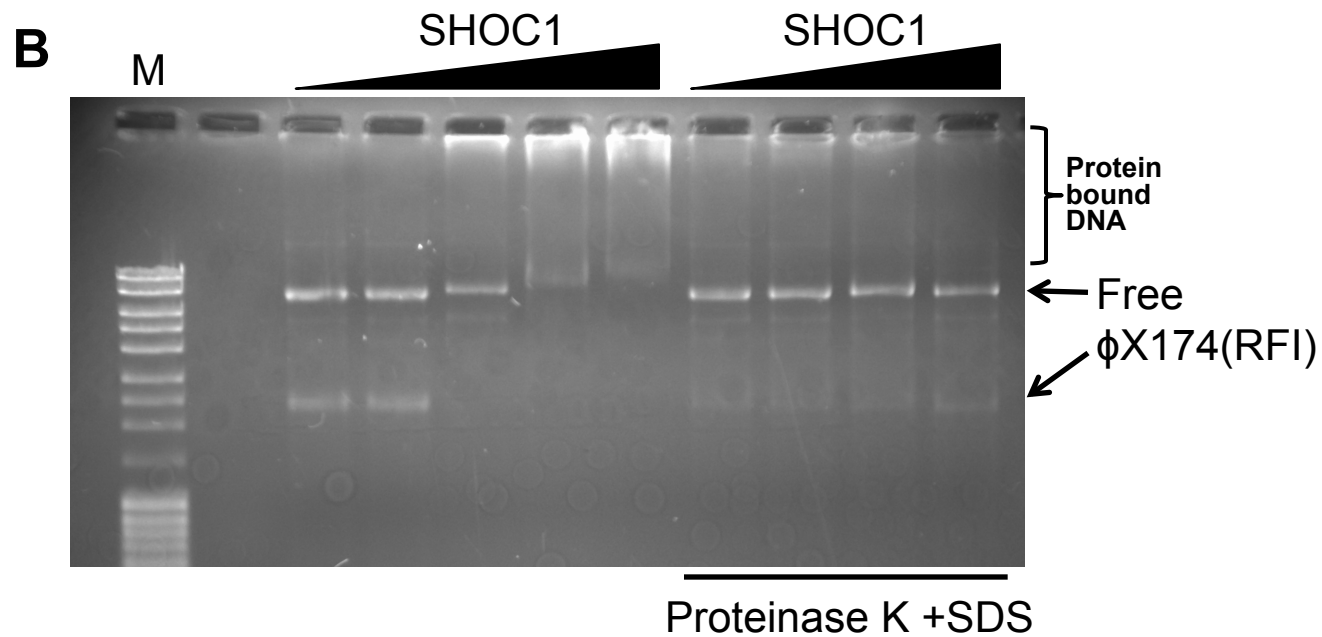

Supplement: S8 Fig — Human recombinant SHOC1570-1111 was incubated with oligonucleotide-based Holliday junction structures and developed in TAE-polyacrylamide gels (A) or supercoiled dsDNA φX174 (RFI) and developed in 1% agarose gels (B) as indicated in Materials and Methods. (PDF) [file pgen.1007381.s008.pdf]

Guiraldelli\_Fig S9

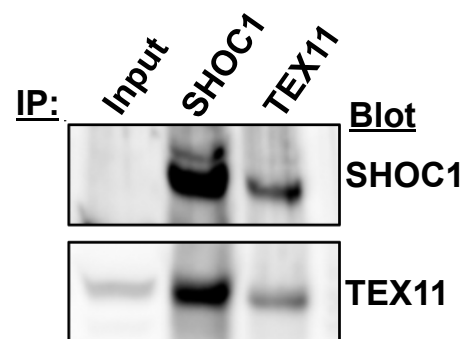

Supplement: S9 Fig — The product of co-immunoprecipitation of TEX11 and SHOC1 from total testis extract of 13 day-old mice is shown. SHOC1, and TEX11 lines are 8X compared to input. Signal acquisition time in the upper and lower panel is not equal. (PDF) [file pgen.1007381.s009.pdf]
