# Supplementary material for: SHOC1 is a ERCC4-(HhH)2-like protein, integral to the formation of crossover recombination intermediates during mammalian meiosis
Source: PLoS Genet. 2018 May 9;14(5):e1007381. doi: 10.1371/journal.pgen.1007381 (PMC5962103; doi:10.1371/journal.pgen.1007381)
Supplement: S3 Fig — (A) Shoc1 gene targeting design for mutants in which 2–21 exons were deleted. (B, C) Genotype analysis of adult mice (B) and 9.5dpc embryos (C) from Shoc1 heterozygous crosses. Note absence of mice carrying knockout genotype. (D) Expression pattern of Shoc1 in selected adult tissues. Tissue distribution expression data were obtained from the Mouse Genomic Informatics (MGI) mouse genome database (MGD) mouse genome database. Ensembl IDs are used for genes. (E) Expression pattern of Shoc1 in selected tissues during embryonic development. Data were obtained from the RNA profiling data sets generated by the mouse ENCODE transcriptome project (BioProject PRJNA66167). CNS, central nervous system. (F) Shoc1 gene targeting design for mutants in which 2–4 exons were deleted. Oligonucleotides and PCR products used for confirmation of vector construction using long distance PCR also shown. (PDF) [file pgen.1007381.s003.pdf]

Guiraldelli_Fig S3

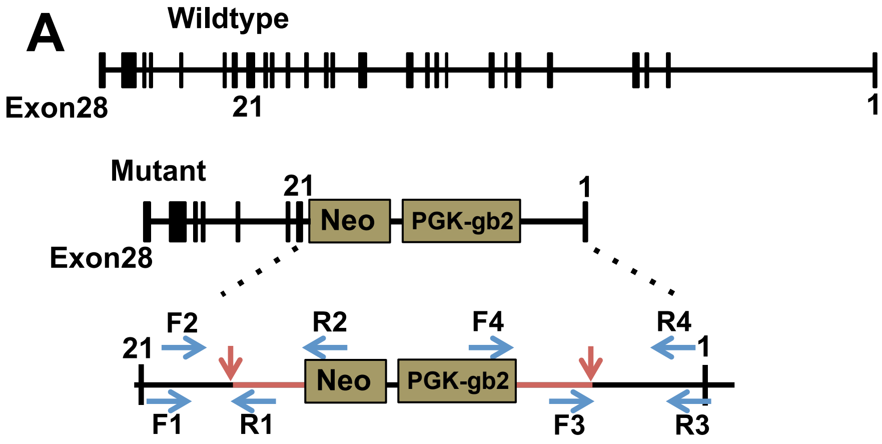

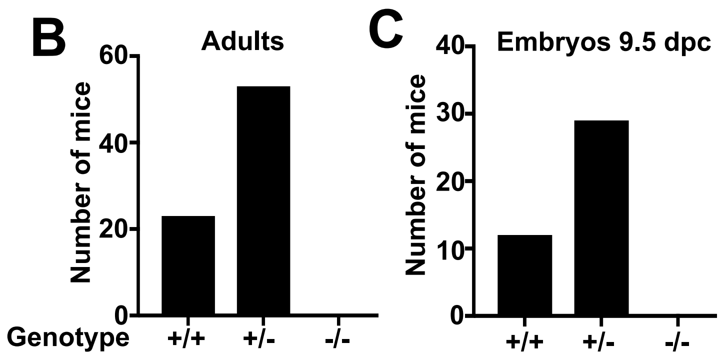

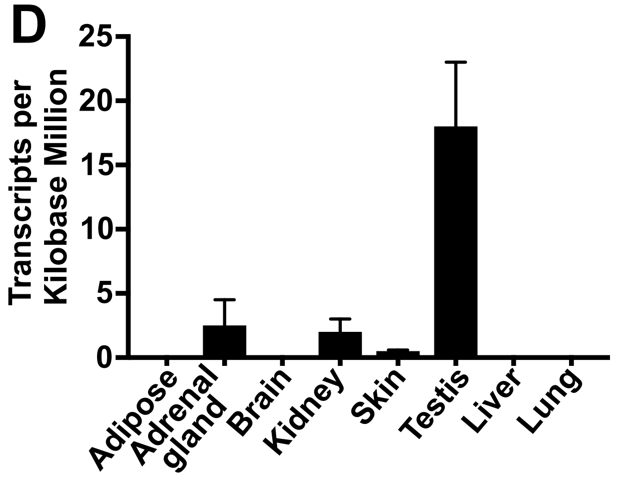

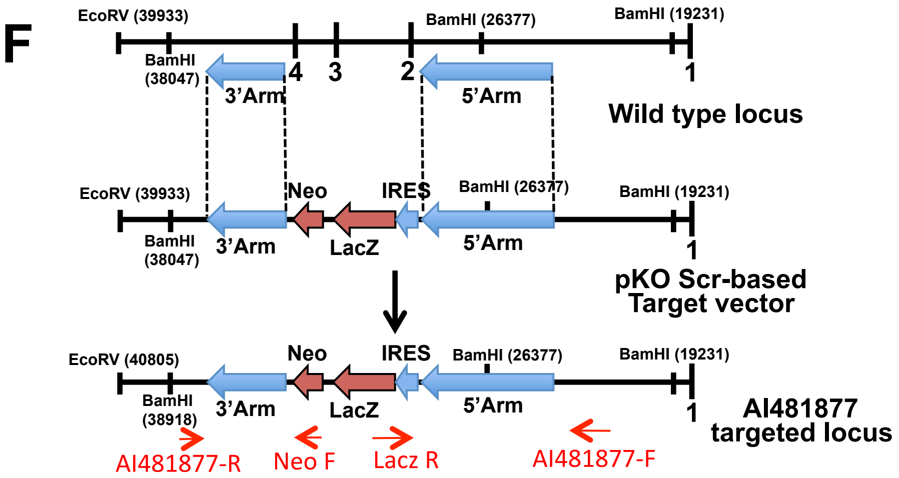

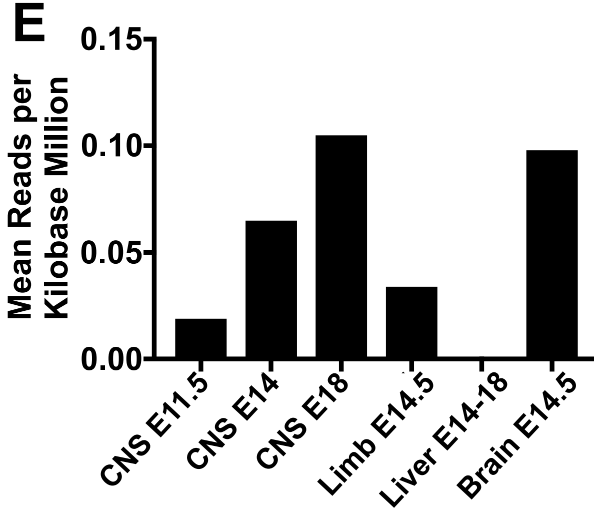

Long distance PCR confirmation

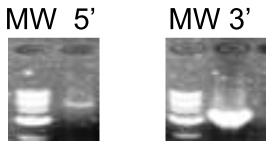

1% Agarose, NEB 1kb ladder

Primer Sequences (5' to 3'):	
5' end PCR: AI481877-F+ (LacZ) IRES R (5.3 kb)<br>3' end PCR: Neo F + AI481877-R (3.8 kb)	
AI481877-F	GCATGCATGTGCCAGCCAGTGTGTA
LacZ R	GGAAAGACCCCTAGGAATGCTCGTCAA
Neo F	GACCGCTTCCTCGTGCTTTACGGTAT
AI481877-R	CCTTGTCTCACAACATGTCAGCGCTT
