# Supplementary material for: SHOC1 is a ERCC4-(HhH)2-like protein, integral to the formation of crossover recombination intermediates during mammalian meiosis
Source: PLoS Genet. 2018 May 9;14(5):e1007381. doi: 10.1371/journal.pgen.1007381 (PMC5962103; doi:10.1371/journal.pgen.1007381)
Supplement: S4 Fig — (A) Shoc1 gene targeting design for mutants in which 2–21 exons were deleted. (B) Results obtained using long PCR-based analysis of the Neo-PGK-gb2 insertion in Shoc1 heterozygous mice. The primers used are indicated in A. (C) Sequence of two PCR fragments (F2-R2, and F4-R4 primers) shown in B. Sequenced portions are underlined (PDF) [file pgen.1007381.s004.pdf]

Guiraldelli_Fig S4

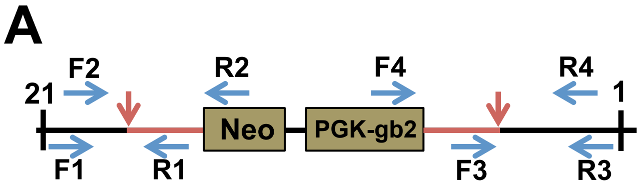


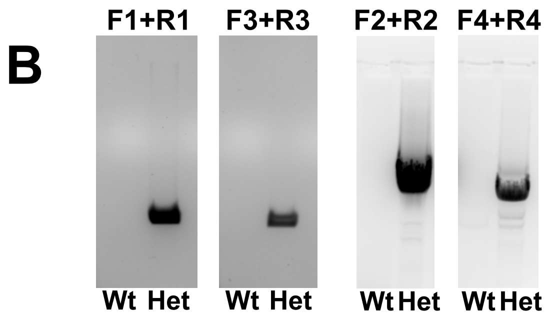


**C**

## F2-R2

TCACTGAGAAATTTACTGAAGAAGGGGGCCAGTTTAGTGTTCCTATCCAAAACTTAGAAACTCATAC
AAGCAGCCAAGCTTCTAAAGTAACCCAAACATTGAAGGTTAAAATAGAAAGGAAATTGTACATCTCT
GGTTAATATTACCATGGTGTTTTTAATATGTAAAGTACATCATATTTCCCAAGTAGATATTCTAGTATAT
AGATTTTCACTATAACTAACTGTGAATCAGCATTGTATTGGTAGCTTTATAATGATGGAGTCAGTGAT
TTCATATGTATGCTTTTTAGATCATATCTATTTACTGTATGTCCTTACAGATTAAATGATGCAGAGAAC
AGAAAGCCAGACTCAGTCAGAGTCCAGTATTTTTATTTCTTTAAACTTTTGGTACTGTTTTGAAGAA
GAGAATACTTCCCTTGATTGATTAAGTCCGTCTTTGTTGATAAAGTATATGTCACTATTATGCAACTG
TTTCAGAACATTTGTCTTTGTTTCCATATGCTAAATAATACTACATTTTATTGAGATTTTCCTTTTGTCT
GGGTACCACATTCTGTGTTTTATATACATAGTATAATAGGGAACTTAATGATTTCAGTGTGTTTGAAAA
ATCTGTTAATATTTAACATCTAGATCTCTGGTATAACTATTAGATTTTTTATTTATTTCTTTTCAGGATTG
CTTATTGATGAGGAAATGATTTTTATGAATAAAGCAATGGACAACCATCTACCAACTGTGAATGGTCT
CCTGAGCAGACTGAAGCTCTATCTGGTGAAGGATCCATTTTTAGATTTCAAAGAAGAGCTCTCTGG
AAAGGATAATTTCACGTATGTCATTCCCAGTGCTGTAACTGCTTTGGTAGCAGAGAACAGTCTTTGT
GTGGCCACAGGGTTAGATGCTGTTGGTGAGGGTTCCAGTGCTTCATCTCAACTTACACGAGAGTC
TTTGTGTGTTTTGATTGGCAGTGCTTATTATGTGTTTTTAGTAAGTATTAATTAATCTTGTTTGATTTTT
TTATTTAAGGGAGTATTTCTCTGTTCAAGAATGTTCGGAACCTTTTGTGAGAGATTTCCATATGGCT
GAGGAGACCTTTTGTAAGAAGAAACTAGTATGTTTCAATAGAATCCTAAACTTTACTTTCAGTAATGT
ATTAGCATACCTTTATAAGTTTTAGTAATAACTATCATGTGAAAATGTTAGTACTAGGTTGAATATGTGT
CAATTATAGAGTCAAATTTACTTCTCAGTAATGAGTTTCAGCCATAGCTGAGGTTGAAGTTGAATCC
AGTTATAGCCTGACTTTATTGGTCTAGTGGGCAGTCCATGTACAGAGAATTTTCAGACCCTTTTAAA
AATTTTAATCACTATACTTGGTATAGAACTTTAAGTAGTTAAGTTTAAAAATTATAAGCTAAATCCTATT
GAATTCTAATTTAAATACACATATAATGTATTGTGGACAAGTGAAACATTTTATCTATTGGGGCTTTTC
TTAATACTTTCAGAGGATATAAACACGAGGCAGTAAAGGCACTTCAAGAATTTTTTTTACAGATAAAA
TGTGAACAACTTGGTAAAGATATTAATTTGCTTATTGAATTCCTGACTCCATCCACTCTCACCTCTCT
TGTTAATTCTGAGTGGTGCAGTCTCACATGTTGAGGA.............ACCTGACGTCCTCTTCTGGAGT
GTCTGAAGACAGCTACAGTGTACTTACATATAATAAATAAACAAATCATTTTAAAAAATCCGTGTTACA
CCCAAGAAATTGATGTGTATATAAGAGTTGTTTATGAGCGTCGTGTGCTACAAAGGCTGAAGTTAAA
GATGAACAGTAAGTAAGGCTGGGAGAGGCCGGCTCAGTAGTGAGCTCAGCAGCTCAAGCAGGGA
GACCCGAGCTTGACCCTCAGCACTCACGGAAAGCCAAGCCTGCCTGTGCAAGCCTGACTCTAGT
GCTGAGAGGACTGGCATAGGAGGCTGTCTGGGCTCACAACCCATCTGCCTAAGTCAAATCAGTGA
GCTGGGGGGTTCAGTGAGCGACTGTGTCTGAAAAATAATGTGGAAAGTGACTAAGGAAGCTCCC
CAACATTGCCCGCTAGCTTCTACGTGCACATATGTGCAGGCACATGCACAGTCATATCCAGAAACA
TGTGCACAGAATAACAACAATAATAAAGTCATAGGCACATATGCCAGGCAACTGGAAAAGGAAATAA
TCCTATCTTAGCTTTCAATCATTTTGCTGATTTTTGCATTTTTACATATTTTGGTGATTTTGATATATGA
GGAATTGAAGGGAGTATGCATTGGAGAATAGGTATTACCTATATGTCAACATTTTTTTCAGAATATAC
ATGACTTTAATGGTACTTTGTTGATAGAGTTTCTTCCATTTCCTTTCAGtcatgacgcgccgcacacaaaaacc
aacacacagatcatgaaaataaagctcttttattggtaccgaattcgccagggagctctcagacgtcgcttggtcggtctttattcgaaccccaga
gtcccgcTCAGAAGAACTCGTCAAGAAGGCGATAGAAGGCGATGCGCTGCGAATCGGGAGCGGCGA
TACCGTAAAGCACGAGGAAGCGGTCAGCCCATTCGCCGCCAAGCTCTTCAGCAATATCACGGGTA
GCCAACGCTATGTCCTGATAGCGGTCCGCCACACCCAGCCGGCCACAGTCGATGAATCCAGAAA
AGCGGCCATTTTCCACCATGATATTCGGCAAGCAGGCATCGCCATGGGTCACGACGAGATCCTCG
CCGTCGGGCATGCGCGCCTTGAGCCTGGCGAACAGTTCGGCTGGCGCGAGCCCCTGATGCTCT
TCGTCCAGATCATCCTGATCGACAAGACCGGCTTCCAT

Red: start subcloning. Green: exons 5 and 6. Blue: Neo reverse orientation.

## F4-R4

gagcccgcccccggacccaccccttcccagcctctgagcccagaaagcgaaggagcaaagctgctattggccgctgccccaaaggcctacccgcttcca
ttgctcagcggtgctgtccatctgcacgagactagtgagacgtgctacttccatttgtcacgtcctgcacgacgcgagctgcggggcggggggaacttcctg
actaggggaggagtggaaggtggcgcgaagggggccaccaaagaacggagccggttggcgcctaccggtggatgtggaatgtgtgcgagccagaggc
cacttgtgtagcgccaagtgcccagcggggctgctaaagcgcatgctccagactgccttgggaaaagcgcctcccctacccggtagaat**ggtacc**GTAA
CATTAATAGTTAATATAACTACTTCCAGTTATGATTTTTAAGAGCTAAAGCTAGCGGCTGGACAGATGGCACAG
TGGTTAATAGCTCTTGCAGAGAACCCACATTGGTTCCCAGCACCCACTTTGGGTAGCTCACACCTCCAGCT
CCTGACCTCCTTTGACATATCCACATGCAGATTCAAACACATATACATAACTACAAATAATAAAAATATTTTAAAA
TGATAGCTCTATACTCAAACTTCAATGAATGGTTTGTTTAATTGATACTCTTAATGTTCTCCATTAACTTTGGTT
TTAAAGGAAAATCAAACTTAGGGTATAACAAAATATTTCAAATTCTTATAGTATCAAATGAACCATACAGTCAAC
TTAAGTTTAAAATAGCTCTCAGGAGGCAGAGACAGGAGGATCTCTATGAGTTCAAGGCCAGCCTGATCTACA
TAGTGAGTTCCAAGACAACCAGGATTACACAGAGAAAACCTGTTGCAAAATAAACAAACAAACAAATAAGAA
AAAGAAGAAATTTAAAAATAGCAAACTTAAGTAAAGCAGAAGATGATATGATTAAACAGAAGAATCAGACTGTT
AAGTATACATATTTATTTGGCTAACATTTGTTAAGCTATTAAGGCTGCTGATGGCTCAGTCACTGTGGTGCTTA
CTGTGCAAGCGTAAGGACATAAGTTTGATACCCAGAAACCATGTGCAAAAGCCTGGCATTGTGGCCATGGC
TCGTGATCCCAGCACTTGGGAGGCAGAAACAGGTGGATTTGTGGGGCTCGATGTCATCCAGCCCAGCTTC
TTTAGTAACACACAGGTGTCTCTCTCTCTCTCTCTCACACACACACACACACACACACACACACACACAC
ACACACACACACTTAAACCTTTTCTATGAGCTGGTTGCCTTTACTGGGTAAGAACCCCTATTATCTTCTCTTT
GATGGATGTTTTTGACTTGACTGAATGTTTTATCATATCGCCTTTTAAAAAGGACAATTTTGTTTTCTCATTTCT
ACTCATTATATCTTTATTTCTATATAATTTTTTATTGCATTGAACATCTAGAACAAAATGTTAAGATCTCATAAGGC
ATATGAATGTAGCGTTGTCATGAAAGGCAGTGGGAGAAGAGAAATTTAAGTTTCAGACTTTCTCTTCTCATAG
TCTTCCTTTCTAAGATCAGAGAAGGGCCCAGAAAAAAAAAAAACTAACATCCATATTTTATTAAAATAGGGCCTC
CTAATCCTGTTTCCACCCTCCCCTCGTTCCTGCCTGCCCTCACCCCTCCTAACGGCTCTGTCTGTTGTTCCC
TGTCCAGTAGACTTCCTTCTGCTTTCATGTCACAGGT..........TTCCATTTGCCAAGGAGCAGCGCCCAACC
TTACTGTTCAAGTCAGACCATTTGCTCATTGGGAAATGAAAAAGCTTTCCTTTACAAAATCAAGGTATCAATTA
TTCCCCAAATTGTACACTTTGTTTCATTTAGTTATTGTAAATTTTTATGAAAAAAATTTTATTAGGGTACAAAAAT
GTTAGAAAAATTACAAATACTCAGTTTTTGGAAATGCAACCTTTCTGTCCTGAAATGCAAAACCCAGTAAATAA
AACATGCCTCTATCCCACACAGGGCTACACAGGCTCCATGGTGAAAGAGGCGCATCCAGCAAGTTTGGTAG
TGAGGTTAGAAGGAAAACATTGACTTCTGTAGGATTTTGACCTAGGACCAGGCCATTTTCTAGGACAGAGAG
AATGACTTGGCTCTTGAAAGCTGTTTTCTTGGGACTTGGCTGCAGTCATCCATCACCATGTGCACACAAGGC
TTCCTCAGCGAGCTTCTTCCCTGTTGACGAGAGTTCCGTTGGTTATCGTCGCTTTAGTGAGAGTTACTTGTA
AAGGAACCTTATCATTTCACAGAACACCAAAAATGCACAATGTAGAAAACATTAGTGAACGCAAGAGAATATG
TAAGCCCATCTTGGTCAGACTGCACTCACAGCTGTGCTATGTAAAGGGGAACTGTGTCACGAAGGCAGTTA
CCTTGGCAACCCAGTTCAACTTGCATCTGATAGCAGAACCAGCCCCCCAATTCAATGTGAACAGTTTCCTTT
TACAAATATGTGAACAAGATGATCACTTACTGTGTAAACAAAATTTGGAATACGTTGGTCCCAGGACTAATAAC
CTATGTCCTACAGTTTTTAGGTTGGTGGGTGGGCAAAAGTTGGAGACTGCTGGATTCACACTGTTTTCACCA
TATCCTACACCAGGGCCAAGCAGGAAGTGAGGAGGTCTGGGTGAGAAGGGCTGCTTCCTCTCCACAACCT
GGGCATACGCACTAGCTGCTTTCCCAACTACCCTGCAGTCTCATTGAGAATGGACAGGATCTATCTCTTACA
ATAGTTTTAATGAAGAGGATTCTTTGATAAACCTTCCCAGAAATTGAAGTGTTTATAGACGAAGGAGCTGCTG
ATTGTTGTCATGAGGGCATCTTGACAGCCATTGGGTAGAAGTGCCCTTACTCAGAACACCAGTAGGCCATGA
GGCTAATCATTTCAAGATCCACTTACCAAGAAAGCCTGAGGTTCTGCTCATCGCTTGAAAATACACCTTCCAT
AAGTCCTCCTTTGACATACTACTTTTTCATGTCAATGGGTATGTGAACAAAATATTTTCCTGATGCTGGCATTT
CAGGGTGAGTAATTCTAAAAGTCTGCATCAGTGCCTGCCAGGGTAACTCACCTTCCCTTCTGAATCCGTTGG
GGCTGGGGTACC

Red: end subcloning. Blue: PGK-gb2 promoters
