# Supplementary material for: SHOC1 is a ERCC4-(HhH)2-like protein, integral to the formation of crossover recombination intermediates during mammalian meiosis
Source: PLoS Genet. 2018 May 9;14(5):e1007381. doi: 10.1371/journal.pgen.1007381 (PMC5962103; doi:10.1371/journal.pgen.1007381)

Guiraldelli_Fig S6

A

```
Homo sapiens      DSDGEKHFLIKILNKIEGLTLTVLHSNERKDFLESEGVLRGTSSCVVVHNQYIGADFPW 802
Gorila gorila     DSDGEKHFLIKILNKIEGLTLTVLHSNERKDFLESEGVLKGTSSCVVVHNQYIGADFPW 766
Pan togrodite     DSDGEKHFLIKILNKIEGLTLTVLHSNERKDFLESEGVLKGISSCVVVHNQYIGADFPW 802
Pongo abelli      DSDGEKHLLIKILNKIEGLTLTVLHSNERKDFLESEGVLKGTSSCVVVHNQYIGADFPW 766
Papio anubis      DSDGEKHLLIQILNKIEGLTLTVLHSKERKDFLESEGVLKGTSSCVVVHNQYIGADFPW 763
Macaca mulata     DSDGEKHLLIQILNKIEGLTLTVLHSKERKDFLESEGVLKGTSSCVVVHNQYIGADFPW 802
Mus musculus      DSDGEKHLLIKTLKKIEGLTMTVLRSNDRKKILETTSILKGTNACVVVHNHSIGADFPW 863
```

B

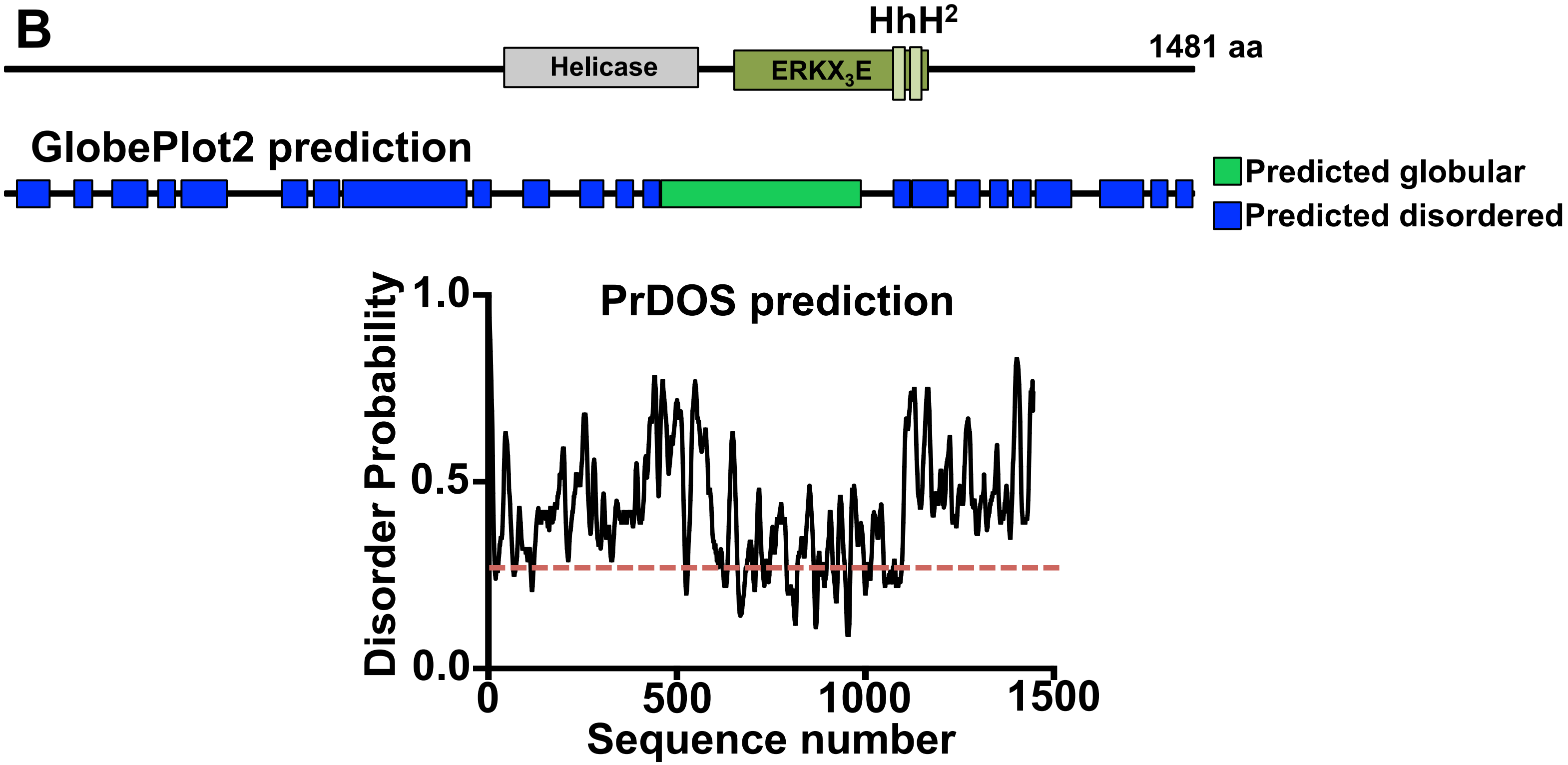

Supplement: S6 Fig — (A) SHOC1 sequence alignment between related mammalian species at the putative Shoc1 XPF-like domain. (B) Prediction of structural disordered areas of human SHOC1 using the GlobePlot2 and PrDOS prediction algorithms. (PDF) [file pgen.1007381.s006.pdf]
