# Supplementary material for: SHOC1 is a ERCC4-(HhH)2-like protein, integral to the formation of crossover recombination intermediates during mammalian meiosis
Source: PLoS Genet. 2018 May 9;14(5):e1007381. doi: 10.1371/journal.pgen.1007381 (PMC5962103; doi:10.1371/journal.pgen.1007381)
Supplement: S1 Table — (PDF) [file pgen.1007381.s010.pdf]

Table S1. Seminiferous tubule size in wild type and Shoc1$^{hyp/hyp}$ testis.

**Wild type**			**Shoc1$^{hyp\backslash hyp}$**		
Diameter 1 (μM)	Diameter 2 (μM)	Average	Diameter 1 (μM)	Diameter 2 (μM)	Average
341	260	300	263	195	229
340	244	292	195	246	220
367	261	314	314	233	273
359	282	320	260	209	234
342	268	305	218	261	239
415	269	342	198	285	241
404	257	330	194	215	204
379	235	322	292	224	258
378	236	307	196	247	221
293	257	275	262	263	262
252	320	286	200	247	223
363	215	289	268	195	365
361	304	332	196	183	287
469	265	366	182	253	308
332	278	305	189	271	227
255	285	270	179	243	211
311	245	278	233	236	234
303	221	262	134	162	148
277	273	325	197	224	210
310	312	311	198	188	193
			240	213	226
			220	216	218
			183	314	248
			203	262	232
			206	262	234
			279	192	235
Average: 342.63	264.57	306.89	217.44	233.76	238.04
		SD: 26.77			40.89
					22.8% reduction
					13% SD
