# Supplementary material for: SHOC1 is a ERCC4-(HhH)2-like protein, integral to the formation of crossover recombination intermediates during mammalian meiosis
Source: PLoS Genet. 2018 May 9;14(5):e1007381. doi: 10.1371/journal.pgen.1007381 (PMC5962103; doi:10.1371/journal.pgen.1007381)
Supplement: S2 Table — (PDF) [file pgen.1007381.s011.pdf]

Table S2. Product of crosses between females wild type and Shoc1$^{hyp/hyp}$ with wild type males.

	Wild type (litter size)					Shoc1hyp/hyp (litter size)				
	Number of pups/litter				total	Number of pups/litter				total
Cage 1	4	5	6	4	19	8	6	8	-	22
Cage 2	7	4	8	7	26	12	4	4	5	25
Cage 3	5	7	5	-	17	8	9	6	-	23
Cage 4	6	9	8	-	23	7	7	6	-	20
