# Supplementary material for: SHOC1 is a ERCC4-(HhH)2-like protein, integral to the formation of crossover recombination intermediates during mammalian meiosis
Source: PLoS Genet. 2018 May 9;14(5):e1007381. doi: 10.1371/journal.pgen.1007381 (PMC5962103; doi:10.1371/journal.pgen.1007381)
Supplement: S3 Table — (PDF) [file pgen.1007381.s012.pdf]

Wild type spermatocytes

Table S3. Measurement of homologous chromosome length in Shoc1$^{hyp/hyp}$ spermatocytes.

Cell1 (µm/ µm/ %)			cell2			cell3			cell4			cell5			cell6			cell7			cell8			cell9			cell10		
8.42	8.42	100.00	9.68	9.68	100.00	11.19	10.39	92.85	5.18	5.18	100.00	6.56	6.56	100.00	9.54	9.54	100.00	9.17	9.17	100.00	7.94	7.94	100.00	7.24	7.24	100.00	9.27	9.27	100.00
6.19	6.19	100.00	7.23	7.23	100.00	12.83	12.46	97.12	8.68	8.68	100.00	5.77	5.77	100.00	7.87	7.87	100.00	8.29	8.29	100.00	7.57	7.57	100.00	9.03	9.03	100.00	9.13	8.51	93.21
8.07	8.07	100.00	7.77	7.77	100.00	10.2	9.29	91.08	4.97	4.97	100.00	8.17	8.17	100.00	8.85	8.85	100.00	7.64	7.64	100.00	9.65	9.65	100.00	7.77	7.51	96.65	9.07	9.07	100.00
7.53	7.53	100.00	8.94	8.94	100.00	6.75	6.75	100.00	9.23	9.23	100.00	7.85	7.85	100.00	9.32	9.32	100.00	10.5	10.5	100.00	4.42	4.42	100.00	14.16	14.16	100.00	8.31	8.31	100.00
5.28	5.28	100.00	11.71	11.67	99.66	13.44	12.43	92.49	6.07	6.07	100.00	7.17	7.17	100.00	9.22	9.22	100.00	11.96	11.96	100.00	10.07	10.07	100.00	10	10	100.00	9.87	9.87	100.00
8.77	8.77	100.00	4.23	4.17	98.58	4.9	4.6	93.88	5.28	5.22	98.86	4.42	4.12	93.21	5.87	4.08	69.51	5.32	5.16	96.99	7.6	7.39	97.24	4.93	4.53	91.89	7.57	7.43	98.15
8.12	8.12	100.00	5.2	5	96.15	4.23	4.15	98.11	4.45	4.35	97.75	7.83	7.71	98.47	6.68	6.62	99.10	2.9	2.7	93.10	5.03	4.95	98.41	9.52	9.43	99.05	9.43	8.91	94.49
6.8	6.8	100.00	8.78	6.61	75.28	5.46	5.1	93.41	6.3	6.01	95.40	5.09	4.91	96.46	6.12	5.96	97.39	5.55	5.47	98.56	6.27	6.16	98.25	5.4	5.21	96.48	9.01	8.62	95.67
8.8	8.8	100.00	5.83	4.8	82.33	5.58	4.4	78.85	5.55	5.47	98.56	6.41	6.31	98.44	6.33	6.19	97.79	5.9	5.9	100.0	4.79	4.73	98.75	4.65	4.64	99.78	6.7	6.31	94.18
8.5	8.5	100.00	5.11	4.82	94.32	4.46	4.26	95.52	4.26	4.09	96.01	6.69	6.46	96.56	7.44	7.41	99.60	5.46	5.39	98.72	7.29	7.28	99.86	4.59	4.32	94.12	5.19	4.83	93.06
average		100.00			99.97			97.35			100.00			100.00			99.28			100.00			99.52			99.00			98.03

Cell11 (µm/ µm/ %)			cell12			cell13			cell14			cell15			cell16			cell17			cell18			cell19			cell20		
6.45	6.45	100.00	7.91	7.91	100.00	9.05	9.05	100.00	6.03	6.03	100.00	6.24	6.24	100.00	8.4	8.4	100.00	8.43	8.09	95.97	6.56	6.56	100.00	5.29	5.29	100.00	8.73	8.73	100.00
7.27	7.27	100.00	9.78	9.78	100.00	9.7	9.36	96.49	6.07	6.07	100.00	7.84	7.84	100.00	9.17	9.17	100.00	6.9	6.9	100.00	6.54	6.54	100.00	7.74	7.74	100.00	12.24	12.24	100.00
11.32	11.32	100.00	15.73	15.73	100.00	9.44	9.44	100.00	7.17	7.17	100.00	7.96	7.96	100.00	9.25	9.25	100.00	9.17	9.17	100.00	8.36	8.36	100.00	5.45	5.45	100.00	7.86	7.86	100.00
7.84	7.84	100.00	11.66	11.66	100.00	14.01	14.01	100.00	7.82	7.82	100.00	6.83	6.83	100.00	9.33	9.33	100.00	7.69	7.69	100.00	7.45	7.45	100.00	5.39	5.39	100.00	9.24	9.24	100.00
10.43	10.43	100.00	8.83	8.83	100.00	10.55	10.55	100.00	6.01	6.01	100.00	7.74	7.74	100.00	8.32	8.32	100.00	9.94	9.94	100.00	9.09	9.09	100.00	7.08	7.08	100.00	5.88	5.88	100.00
8.7	8.7	100.00	8.64	8.64	100.00	5.64	5.2	92.20	10.75	10.75	100.00	6.94	6.94	100.00	10.39	10.39	100.00	12.34	12.34	100.00	7.22	7.22	100.00	7.79	7.79	100.00	9.39	9.39	100.00
8.49	8.49	100.00	9.13	9.13	100.00	7.35	7.35	100.00	8.09	8.09	100.00	6.67	6.67	100.00	9.54	9.54	100.00	11.04	11.04	100.00	5.68	5.68	100.00	7.11	7.11	100.00	9.79	9.79	100.00
6.68	6.68	100.00	6.82	6.82	100.00	11.48	11.48	100.00	7.34	7.34	100.00	9.39	9.39	100.00	7.73	7.73	100.00	4.75	4.75	100.00	8.42	8.42	100.00	7.12	7.12	100.00	8.59	8.59	100.00
8.51	8.51	100.00	7.55	7.55	100.00	14.2	14.2	100.00	7.48	7.48	100.00	8.55	8.55	100.00	8.78	8.78	100.00	9	9	100.00	7.45	7.45	100.00	7.46	7.46	100.00	8.51	8.51	100.00
8.97	8.97	100.00	9.2	9.2	100.00	6.39	6.39	100.00	8.13	8.13	100.00	9.25	9.25	100.00	7.84	7.84	100.00	8.37	8.18	97.73	5.27	5.27	100.00	10.13	10.13	100.00	10.1	10.1	100.00
average		100.00			100.00			98.87			100.00			100.00			100.00			99.37			100.00			100.00			100.00

Shoc1$^{hyp/hyp}$ spermatocytes

Cell1 (µm/ µm/ %)			cell2			cell3			cell4			cell5			cell6			cell7			cell8			cell9			cell10		
4.84	4.5	92.98	3.42	3.09	90.35	4.61	4.23	91.76	4.2	3.92	93.33	6.71	6.65	99.11	4.83	4.7	97.31	3.83	3.79	98.96	4.78	4.58	95.82	6.66	6.63	99.55	8.07	7.94	98.39
3.1	2.79	90.00	4.45	3.45	77.53	5.24	5.15	98.28	4.49	3.59	79.96	6.64	6.51	98.04	5.9	5.9	100.0	6.65	6.52	98.05	6.27	6.06	96.65	5.85	5.77	98.63	6.31	6.08	96.35
4.84	4.34	89.67	6.12	5.95	97.22	6.77	6.54	96.60	3.4	3.01	88.53	7.14	7.06	98.88	4.73	4.72	99.79	4.57	4.54	99.34	6.26	6.11	97.60	5.29	5.26	99.43	6.27	6.26	99.84
6.42	6.32	98.44	6.81	6.45	94.71	5.78	5.74	99.31	3.73	3.56	95.44	5.24	5.14	98.09	4.53	4.49	99.12	5.23	5.06	96.75	7.8	7.75	99.36	6.88	6.71	97.53	8.13	8.08	99.38
5.76	5.62	97.57	4.83	4.83	100.0	6.4	6.15	96.09	6	5.82	97.00	3.41	3.4	99.71	5.84	5.59	95.72	4.99	4.89	98.00	4.56	4.46	97.81	5.6	5.36	95.71	6.9	6.87	99.57
4.38	3.96	90.41	4.23	4.17	98.58	4.9	4.6	93.88	5.28	5.22	98.86	4.42	4.12	93.21	5.87	4.08	69.51	5.32	5.16	96.99	7.6	7.39	97.24	4.93	4.53	91.89	7.57	7.43	98.15
5.39	5.11	94.81	5.2	5	96.15	4.23	4.15	98.11	4.45	4.35	97.75	7.83	7.71	98.47	6.68	6.62	99.10	2.9	2.7	93.10	5.03	4.95	98.41	9.52	9.43	99.05	9.43	8.91	94.49
7.41	7.31	98.65	8.78	6.61	75.28	5.46	5.1	93.41	6.3	6.01	95.40	5.09	4.91	96.46	6.12	5.96	97.39	5.55	5.47	98.56	6.27	6.16	98.25	5.4	5.21	96.48	9.01	8.62	95.67
3.81	3.8	99.74	5.83	4.8	82.33	5.58	4.4	78.85	5.55	5.47	98.56	6.41	6.31	98.44	6.33	6.19	97.79	5.9	5.9	100.0	4.79	4.73	98.75	4.65	4.64	99.78	6.7	6.31	94.18
6.6	6.29	95.30	5.11	4.82	94.32	4.46	4.26	95.52	4.26	4.09	96.01	6.69	6.46	96.56	7.44	7.41	99.60	5.46	5.39	98.72	7.29	7.28	99.86	4.59	4.32	94.12	5.19	4.83	93.06
average		94.76			90.65			94.18			94.08			97.70			95.53			97.85			97.97			97.22			96.91

Cell11 (µm/µm/%)			cell12			cell13			cell14			cell15			cell16			cell17			cell18			cell19			cell20		
8.01	7.53	94.01	4.71	4.32	91.72	6.52	6.49	99.54	6.74	6.61	98.07	8.57	8.43	98.37	9.81	8.45	86.14	7.56	6.61	87.43	5.96	5.92	99.33	6.57	6.53	99.39	7.08	6.73	95.06
4.88	4.75	97.34	5.76	5.75	99.83	4.12	4.11	99.76	3.84	3.67	95.57	7.3	6.62	90.68	10.35	10.24	98.94	3.71	3.52	94.88	5.91	5.73	96.95	4.31	4.28	99.30	8.97	8.97	100.0
7.31	7.24	99.04	6.98	6.88	98.57	5.87	5.51	93.87	4.27	4.08	95.55	4.59	4.37	95.21	10.02	9.62	96.01	5.05	4.63	91.68	4.73	4.56	96.41	7.7	7.55	98.05	14.97	14.6	97.53
5.74	5.7	99.30	6.02	5.89	97.84	3.23	3.21	99.38	4.33	4.09	94.46	4.36	3.63	83.26	6.61	6.5	98.34	5.28	5.13	97.16	5.44	4.37	80.33	4.83	4.58	94.82	5.87	5.34	90.97
6.25	6.15	98.40	6.23	6.13	98.39	5.17	4.93	95.36	3.84	3.56	92.71	6.98	6.62	94.84	6.47	5.98	92.43	5.66	4.77	84.28	4.78	4.77	99.79	7.23	7.2	99.59	8.64	7.87	91.09
5.21	4.99	95.78	6.9	6.74	97.68	5.24	5.03	95.99	5.74	5.59	97.39	8.06	7.99	99.13	3.46	3.25	93.93	7.3	7.24	99.18	3.97	3.92	98.74	3.26	3.16	96.93	6.27	6.2	98.88
4.87	4.7	96.51	6.94	6.56	94.52	6.93	6.83	98.56	4.98	4.82	96.79	5.27	5.18	98.29	12.26	11.81	96.33	6.89	6.73	97.68	6.88	6.37	92.59	4.4	4.04	91.82	7.07	6.9	97.60
6.8	6.38	93.82	8.13	7.8	95.94	6.18	6.14	99.35	6.58	6.53	99.24	6.7	6.17	92.09	7.17	7.02	97.91	8.1	7.38	91.11	5.76	5.31	92.19	6.69	6.62	98.95	5.48	5.08	92.70
5.88	5.58	94.90	3.14	3.03	96.50	5.34	4.99	93.45	5.41	5.34	98.71	8.6	7.88	91.63	8.68	8.57	98.73	5.91	5.07	85.79	5.24	5.05	96.37	5.74	5.61	97.74	5.32	5.05	94.92
6.43	6.35	98.76	5.22	5.16	98.85	4.47	4.23	94.63	5.4	5.25	97.22	5.7	5.02	88.07	7.63	7.59	99.48	8.98	8.45	94.10	6.88	6.49	94.33	5.84	5.53	94.69	4.32	3.74	86.57
average		96.79			96.98			96.99			96.57			93.16			95.82			92.33			94.70			97.13			94.53
