# Supplementary material for: SHOC1 is a ERCC4-(HhH)2-like protein, integral to the formation of crossover recombination intermediates during mammalian meiosis
Source: PLoS Genet. 2018 May 9;14(5):e1007381. doi: 10.1371/journal.pgen.1007381 (PMC5962103; doi:10.1371/journal.pgen.1007381)
Supplement: S4 Table — (PDF) [file pgen.1007381.s013.pdf]

Table S4. Oligonucleotides used in this study.

Primer	Application	Forward (5’ to 3’)	Reverse (5’ to 3’)
Primer F2	Genotyping Shoc1^{hyp}	GGCCACAGCTATTCCAGTTC	
Primer Neo	Genotyping Shoc1^{hyp}		GACCGCTTCCTCGTGCTTTACGGTAT
Primer R1	Genotyping Shoc1^{hyp}	GCAAATATCTGAAGGGTGACC	
Primer 5’F	Genotyping Shoc1KO	GAAGGGAGTATGCATTGGAG	
Primer R2	Genotyping Shoc1KO		GAAGTCAGTGACAGGCGAAA
Primer SAPR3	Genotyping Shoc1KO	TCTGGATTCATCGACTGTGG	
Exons 2-3	qPCR-Shoc1 expression	ATTGGACTTTGTATGGCATTGA	AAGCATCTCTGTAAAGCCTCTC
Exons 3-4	qPCR-Shoc1 expression	AGAGAGGCTTTACAGAGATGC	GTTCTCCACTGCTCCAAGAC
Exons 4-5	qPCR-Shoc1 expression	ATTCAGTCTTGGAGCAGTGG	AGGTGGAGAGTTTGGATTGG
Exons 5-6	qPCR-Shoc1 expression	GTGTTCCTTGTACTACCCCG	CCTTCACCAGATAGAGCTTCAG
Exons 8-9	qPCR-Shoc1 expression	TGGACTCTGTAGATGAAAGTGAATG	TGGCTCACTGCACTCTGGTTC
Exons 11-12	qPCR-Shoc1 expression	GGGTCTCGTTCTTGCTTACAG	GATGTAGTCGTGCTTGTCTCC
Exons 22-23	qPCR-Shoc1 expression	TGGGTCACTGAAACTCTTTGG	GAACGACAGTGCCATTAATCTC

Primer	Application	Forward (5’ to 3’)
#1	DNA binding	ACGCTGCCGAATTCTACCAGTGCCTTGCTAGGACATCTTTGCCCACCTGCAGGTTCACCC (60bp)
#2	DNA binding	GGGTGAACCTGCAGGTGGGCAAAGATGTCCTAGCAAGGCACTGGTAGAATTCGGCAGCGT (60bp)
#3	DNA binding	ACGGCATAAAGCTTGACGATTACAACAGATCATGGAGCTGTCTAGAGGATCCGACTATCG (60bp)
#4	DNA binding	CGATAGTCGGATCCTCTAGACAGCTCCATGTAGCAAGGCACTGGTAGAATTCGGCAGCGT (60bp)
#5	DNA binding	GGGTGAACCTGCAGGTGGGCAAAGATGTCCTAGCAAGGCACTGGTAGAATTCGGCAGCGT (60bp)
#6	DNA binding	GGGTGAACCTGCAGGTGGGCAGGAAGGCAAAATGCCGCAACTGGTAGAATTCGGCAGCGT (60bp)
#7	DNA binding	TTGCGGCATTTTGCCTTCCTCGGCCAGTGAATTCGAGCTC (40bp)
